# The Efficacy of Antioxidative Stress Therapy on Oxidative Stress Levels in Rheumatoid Arthritis: A Systematic Review and Meta-analysis of Randomized Controlled Trials

**DOI:** 10.1155/2021/3302886

**Published:** 2021-10-07

**Authors:** Liuting Zeng, Ganpeng Yu, Kailin Yang, Jun Li, Wensa Hao, Hua Chen

**Affiliations:** ^1^Department of Rheumatology and Clinical Immunology, Peking Union Medical College Hospital, Chinese Academy of Medical Sciences & Peking Union Medical College, Beijing, China; ^2^People's Hospital of Ningxiang City, Ningxiang City, Hunan Province, China; ^3^Beijing Anzhen Hospital, Capital Medical University, Beijing, China; ^4^Institute of Materia Medica, Chinese Academy of Medical Sciences and Peking Union Medical College, Beijing, China

## Abstract

**Objective:**

To explore the efficacy of antioxidative stress therapy on oxidative stress levels in rheumatoid arthritis (RA) by a systematic review and meta-analysis of randomized controlled trials.

**Methods:**

Chinese and English databases such as PubMed, Embase, China National Knowledge Infrastructure (CNKI), and China Biomedical Literature were searched, mainly searching for clinical randomized controlled trials of antioxidant therapy for rheumatoid arthritis. The search time is from the establishment of the database to July 2021. Two researchers independently carried out literature search, screening, and data extraction. The bias risk tool provided by the Cochrane Collaboration was used to evaluate the bias risk of all the included literature, and the RevMan 5.3 software was used for meta-analysis.

**Results:**

A total of 24 RCTs (28 records) and 1277 participants were included. The time span of randomized controlled trials (RCTs) is from 1986 to 2020. These RCTs involve 14 types of antioxidants or antioxidant therapies, and these therapies have varying degrees of improvement on oxidative stress in RA patients. The summary results showed that the MDA in the experiment group is lower (SMD -0.82, 95% CI -1.35 to -0.28, *P* = 0.003). The difference of TAC, SOD, NO, GPx, CAT, and GSH between two groups was of no statistical significance (TAC (SMD 0.27, 95% CI -0.21 to 0.75, *P* = 0.27), SOD (SMD 0.12, 95% CI -0.16 to 0.40, *P* = 0.41), NO (SMD -2.03, 95% CI -4.22 to 0.16, *P* = 0.07), GPx (SMD 0.24, 95% CI -0.07 to 0.54, *P* = 0.13), CAT (SMD 2.95, 95% CI -2.6 to 8.51, *P* = 0.30), and GSH (SMD 2.46, 95% CI -0.06 to 4.98, *P* = 0.06)). For adverse events, the summary results showed that the difference was of no statistical significance (RR 1.16, 95% CI 0.79 to 1.71, *P* = 0.45). In addition, antioxidant therapy has also shown improvement in clinical efficacy indexes (number of tender joints, number of swollen joints, DAS28, VAS, and HAQ) and inflammation indexes (ESR, CRP, TNF-*α*, and IL6) for RA patients.

**Conclusion:**

The existing evidence shows potential benefits, mainly in reducing MDA and increasing TAC and GSH in some subgroups. However, more large samples and higher quality RCTs are needed to provide high-quality evidence, so as to provide more clinical reference information for the antioxidant treatment of RA.

## 1. Introduction

Rheumatoid arthritis (RA) is a chronic systemic autoimmune disease of unknown etiology [1]. In the United States, RA affects more than 1.3 million adults, accounting for 0.6%–1% of the population [1, 2]. Epidemiological research shows that the prevalence of rheumatoid arthritis in China is 0.2%~0.36%, which has increased from 5.8 million cases in 2015 to 5.9 million cases in 2019, and the 3-year disability rate has reached 70%; it has become a serious public health problem [3, 4]. The clinical manifestation of RA is mainly a chronic inflammatory (nonsuppurative inflammation) disease of peripheral multiple joints. It may be accompanied by extra-articular systemic damage (causing subcutaneous nodules, pericarditis, myocarditis, pulmonary fibrosis, pleurisy, splenomegaly, renal amyloidosis, peripheral neuritis, arteritis, etc.) [5]. The pathological features of RA are mainly manifested as synovitis of the joints (which can later spread to articular cartilage, bone tissue, joint ligaments, and tendons), followed by extensive inflammation of connective tissues such as serosa, heart, lung, and eyes [5, 6]. When the disease involves cartilage and bone, joint deformities may occur, that is, synovial inflammation, exudation, cell proliferation, granuloma formation, cartilage and bone tissue destruction, and finally joint stiffness and dysfunction [6]. The cartilage destruction of joints is related to the abnormal expression of cytokines, and the imbalance between protective cytokines and destructive cytokines is the basis of RA pathology [7]. In addition, inflammatory chemokines and immune-inflammatory cells jointly promote the exacerbation of the pathological process of RA [8].

Current research shows that in addition to inflammation [9], oxidative stress products also play an important role in the pathogenesis and pathological progress of RA [10]. Oxidative stress can produce too many free radicals, which will cause the oxidation of many molecules in the body. Excessive free radicals in the body of RA patients increase the level of the oxidation marker malondialdehyde (MDA), and the antioxidant enzyme superoxide dismutase (SOD) system is disturbed, which leads to the weakening of the body's antioxidant capacity and aggravating bone destruction [11–14]. In addition, oxidative stress is closely related to the energy metabolism of synovial tissue in RA patients [15]. Therefore, research on oxidative stress, SOD antioxidation, and regulation relationship in patients with RA can reveal the pathological mechanism of RA and find new anti-RA drugs. At present, many randomized controlled trials (RCTs) of antioxidants [16–20] in the treatment of RA patients have been published. However, the results and interventions of these RCTs are diverse, and the quality of the evidence provided varies, which cannot provide clinical doctors with evidence to formulate treatment measures against oxidative stress. Therefore, it is urgent to conduct a comprehensive and in-depth systematic review and meta-analysis of these RCTs for the treatment of RA against oxidative stress. Therefore, this study will conduct a comprehensive systematic review and meta-analysis of RCTs for the treatment of RA against oxidative stress for the first time, in order to provide clinicians with high-quality evidence and promote the clinical practice of antioxidant treatment of RA in the future and to further improve the adjuvant therapy for RA patients.

## 2. Why Is This Systematic Review Important?

Oxidative stress plays a central role in the pathogenesis of RA. At present, evidence of clinical randomized controlled trials surrounding oxidative stress interventions has been reported one after another. However, the results and interventions of these RCTs are diverse, and the quality of the evidence provided is not uniform, and the levels are not uniform, which cannot provide clinical doctors and patients with evidence and treatment measures for the pathological mechanism of oxidative stress. Therefore, it is urgent to conduct a comprehensive and in-depth systematic review and meta-analysis of these RCTs for antioxidative stress treatment, in order to provide clinicians with high-quality evidence in the future, promote the clinical practice of RA treatment, and further improve the adjuvant treatment measures of RA.

## 3. Materials and Methods

### 3.1. Protocol

This systematic review and meta-analysis was conducted strictly in accordance with the protocol registered in PROSPERO (CRD42021256587) and PRISMA guidelines (see Supplementary Materials (available here)) [21].

### 3.2. Literature Search Strategy

English databases and Chinese databases were searched with the retrieval time up to July 2021. English databases include PubMed, Embase, MEDLINE Complete, Web of Science, and Cochrane Library. Chinese databases include Wanfang Database on Academic Institutions in China, China National Knowledge Infrastructure (CNKI), VIP Database for Chinese Technical Periodicals, and China Biology Medicine (CBM). This study also searched the Cochrane Library and ClinicalTrials.gov. The search strategy of PubMed and Embase is shown in Table S1 as an example.

### 3.3. Inclusion and Exclusion Criteria

#### 3.3.1. Participants

Participants are RA patients. The diagnosis of RA conforms to the RA diagnostic criteria in the 2010 Rheumatoid Arthritis Diagnostic and Treatment Guidelines of the Chinese Medical Association Rheumatology Branch or the standard RA diagnostic criteria proposed by the American Academy of Rheumatology in 1987/European Rheumatism League in 2017 or other recognized diagnostic criteria for RA.

#### 3.3.2. Intervention

The treatment of the experimental group is antioxidative stress therapy with no limitations to forms, preparations, and so on; the therapy could be combined with conventional therapy or the therapy in the control group. The treatment of the control group was conventional therapy or placebo or other nonantioxidative stress therapies.

#### 3.3.3. Outcomes

The outcomes were clinical efficacy indexes, inflammation indexes, adverse events, and oxidative stress-related indicators. Clinical efficacy indexes include the number of tender joints, number of swollen joints, 28-joint disease activity score (DAS28), Health Assessment Questionnaire (HAQ), and Visual Analog Scale (VAS); inflammation indexes include erythrocyte sedimentation rate (ESR), C-reactive protein (CRP), tumor necrosis factor- (TNF-) *α*, and Interleukin- (IL-) 6; oxidative stress-related indicators include malondialdehyde (MDA), glutathione (GSH), Catalase (CAT), glutathione peroxidase (GPx), nitric oxide (NO), superoxide dismutase (SOD), and total antioxidant capacity (TAC).

#### 3.3.4. Study Design

The study design includes randomized controlled trials (RCTs), with no limitations to publication time, language, quality, and publication status.

#### 3.3.5. Exclusion Criteria

Exclusion criteria include non-RCT, review, cohort study, and patients with other rheumatism (such as systemic lupus erythematosus and Sjogren's syndrome).

### 3.4. Literature Screening and Risk of Bias Assessment

The two researchers jointly formulate a literature search strategy, independently collect literature, read literature titles and abstracts, and conduct preliminary screening. Then, the two researchers read the full text of the selected literature and finally determined the literature that met the inclusion criteria. The Cochrane Risk Bias Assessment Form is used to systematically evaluate the quality of the included literature. If opinions are inconsistent, they are resolved through discussion. The content of the risk assessment of bias includes [22] (1) random allocation method, (2) allocation plan hiding, (3) blind method, (4) completeness of result data, (5) selective reporting of research results, and (6) other sources of bias.

### 3.5. Data Extraction

The two researchers independently extracted data from the included literature, filled in the data extraction form, and cross-checked. The extracted content includes general information of the literature (such as author, sample size, patient's age, intervention time, and frequency) and related efficacy evaluation indicators [23].

### 3.6. Statistical Analysis

The Review Manager 5.3 software was used for statistical analysis. Subgroup analysis was carried out according to the intervention measures of RCTs. A heterogeneity test was performed on the included literature. If *I*^2^ > 50% and *P* < 0.1, it is considered that there is a large heterogeneity, and the source of the heterogeneity is analyzed. If *I*^2^ < 50% and *P* > 0.1, the heterogeneity is considered low (i.e., RCTs are homogeneous). The random effect model was used for analysis. For continuous variables, if the indicator units or measurement methods were different, or the value differs by more than 10 times, standardized mean difference (SMD) and 95% confidence interval (CI) would be used as the effect size indicator; for indicators with the same unit, weighted mean difference (WMD) and 95% confidence interval (CI) were used as the effect size indicator. For dichotomous variables, the risk ratio (RR) and 95% CI were used as the effect size indicator [23]. The publication bias was detected by STATA 15 with the Egger method (continuous variable) for outcomes with more than 5 RCTs. *P* > 0.1 is considered to have no publication bias.

## 4. Results

### 4.1. Results of the Search

**Figure 1:**
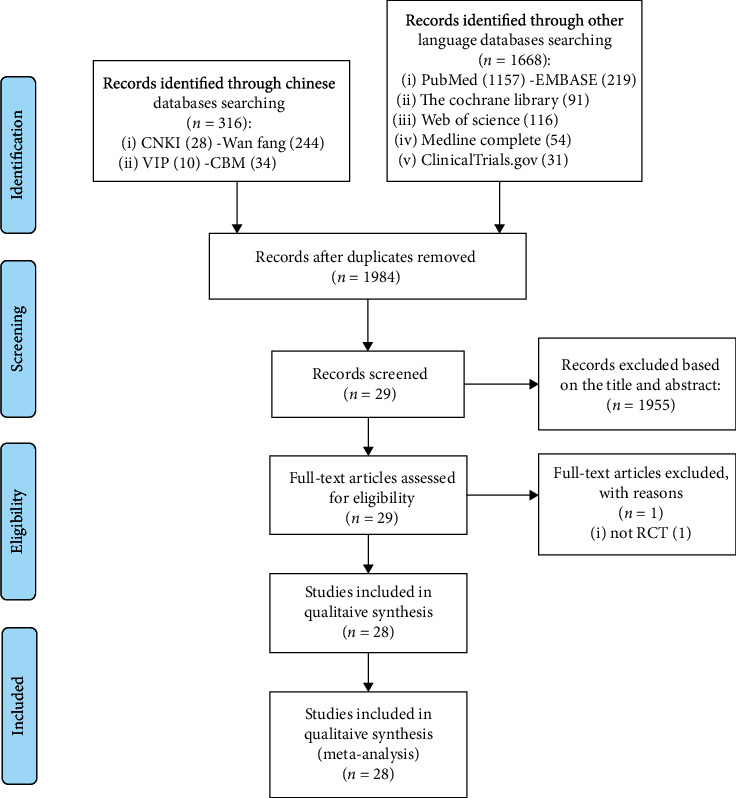
Flow diagram.

The total records identified through database searching and other sources were 1984. According to the search strategy, a total of 29 articles were obtained through preliminary search. By eliminating duplicate documents and carefully reading the title and abstract, a total of 1955 articles were excluded. After carefully reading the full text and comparing the selection criteria, 28 records were screened out and finally included (Figure 1).

### 4.2. Description of Included Trials

Among the 28 records, 2 records [19, 20] belong to Abdollahzad et al. 2015, 2 records [24, 25] belong to Javadi et al. 2017 [24, 25], 2 records [26, 27] belong to Moosavian et al. 2020, and 2 records [28, 29] belong to Mirtaheri et al. 2015; therefore, a total of 24 RCTs and 1277 participants (most of them are female) were included. The time span of RCTs is from 1986 to 2020. Among those RCTs, 3 RCTs utilized N-acetylcysteine [16–18]; 2 RCTs utilized CoQ10 [19, 20, 30, 31]; 2 RCTs utilized probiotic [31, 32]; Ghavipour et al. 2016 utilized pomegranate extract [33]; 2 RCTs utilized quercetin [24, 25, 34]; Khojah et al. 2018 utilized resveratrol [35]; Moosavian et al. 2020 utilized garlic tablets [26, 27]; Aryaeian et al. 2009 [36] utilized conjugated linoleic acids, conjugated linoleic acids plus vitamin E, and vitamin E; 3 RCTs utilized vitamin E [36–38]; 4 RCTs utilized selenium [39–42]; Karagülle et al. 2017 utilized spa therapy [43]; Jaswal et al. 2003 utilized vitamins A, E, and C combination [44]; León Fernández et al. 2016 utilized ozone [45]; Ishibashi et al. 2014 utilized H_2_-saline [46]; and 2 RCTs utilized alpha-lipoic acid [28, 29, 34]. Among those RCTs, 7 RCTs were registered clinical trials. Two RCTs were from Belgium; 2 RCTs were from China; 2 RCTs were from Germany; 8 RCTs were from Iran; Bae et al. 2009 was from Korea; Khojah et al. 2018 was from Egypt; Edmonds et al. 1997 was from the UK; Tarp et al. 1986 was from Denmark; Karagülle et al. 2017 was from Turkey; Jaswal et al. 2003 was from India; León Fernández et al. 2016 was from Cuba; and Ishibashi et al. 2014 was from Japan. Bae et al. 2009 [34] contains two intervention methods, so they were divided into Bae et al. 2009a and Bae et al. 2009b. Aryaeian et al. 2009 [36] has 3 intervention methods, so they were divided into Aryaeian et al. 2009a, Aryaeian et al. 2009b, and Aryaeian et al. 2009c. The details of study characteristics are presented in Table 1.

### 4.3. Risk of Bias Assessment

**Figure 2:**
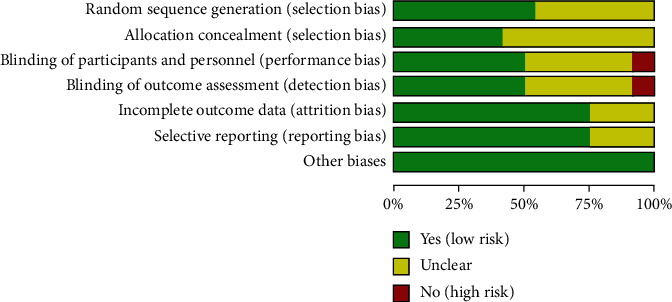
Risk of bias graph.

**Figure 3:**
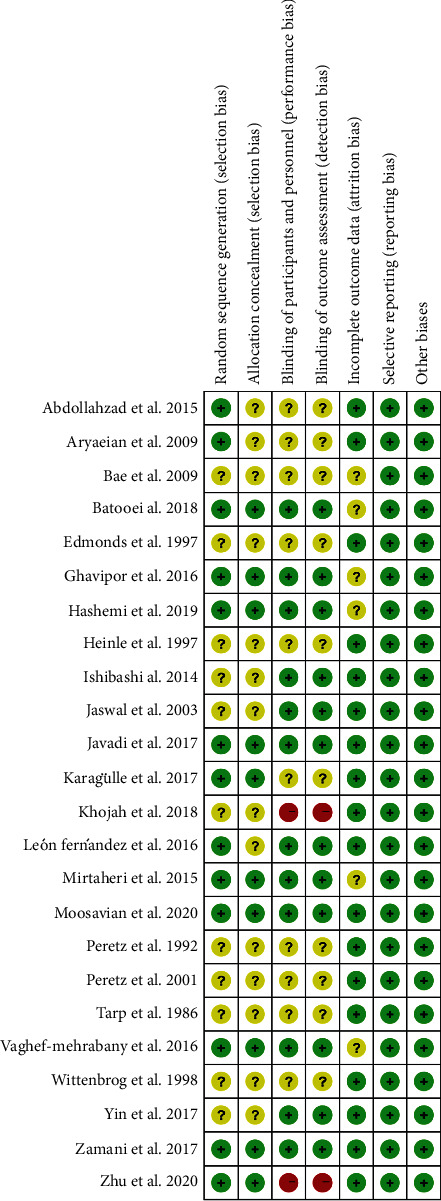
Risk of bias summary.

The RCTs were assessed by “risk of bias” assessment tools. The summary and graph of risk of bias are shown in Figures 2 and 3.

#### 4.3.1. Random Sequence Generation and Allocation Concealment

Thirteen (13) RCTs describe random sequence generation methods [16, 17, 19, 20, 24–33, 36, 43, 45] and were rated as low risk of bias. The other RCTs do not describe random sequence generation methods and were rated as unclear risk of bias. Fourteen RCTs [18–20, 34–42, 44–46] did not describe allocation concealment methods and were assessed as unclear risk of bias.

#### 4.3.2. Blinding, Incomplete Outcome Data, and Selective Reporting

Only 6 RCTs [16, 17, 26, 27, 31–33] describe the implementation process of the blind method and were rated as low risk of bias. Four RCTs [18, 24, 25, 44, 46] did not describe the implementation process of blinding, and the indicators of this study are biochemical indicators (such as MDA); they are assessed as low risk of bias. Twelve (12) RCTs [20, 28, 29, 34, 36–43, 45] claimed to use blinding but did not describe the implementation process of blinding and included subjective indicators (such as DAS28 and VAS), so they were assessed as unclear risk of bias. Two RCTs [30, 35] did not utilize blinding, and the indicators of this study are subjective indicators (such as VAS and DAS28); they are assessed as high risk of bias. Six RCTs [16, 17, 28, 29, 31, 33, 34] have missing data, and the number of missing is unbalanced, but no appropriate statistical treatment method is specified, and the risk of bias is estimated to be unclear.

### 4.4. Other Potential Bias

Other sources of bias were not observed in 24 RCTs; therefore, the risks of other bias of the RCTs were low.

### 4.5. Outcomes

**Figure 4:**
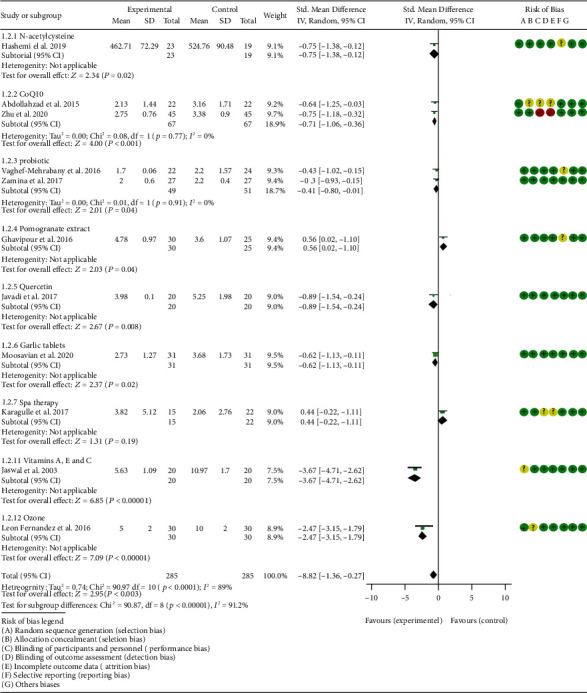
MDA.

**Figure 5:**
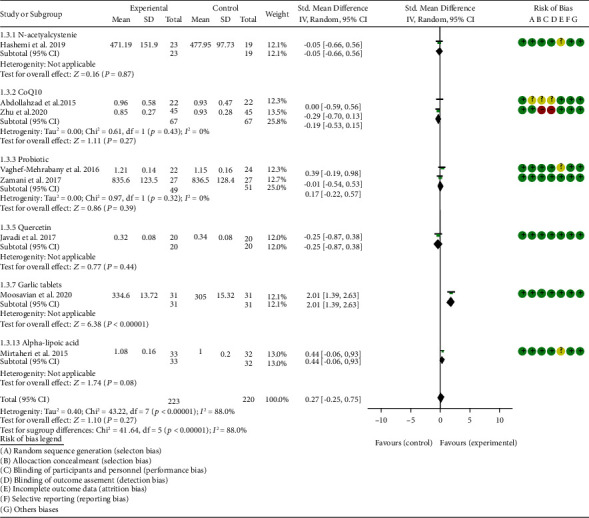
TAC.

**Figure 6:**
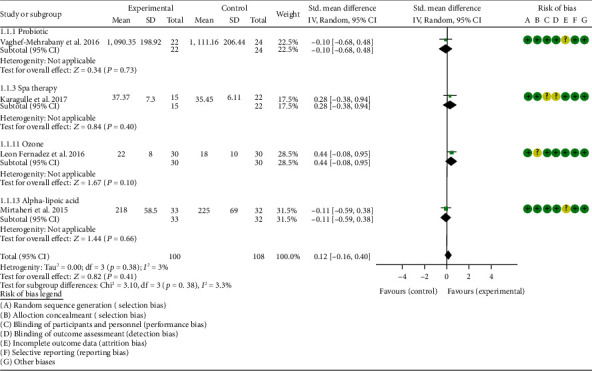
SOD.

**Figure 7:**
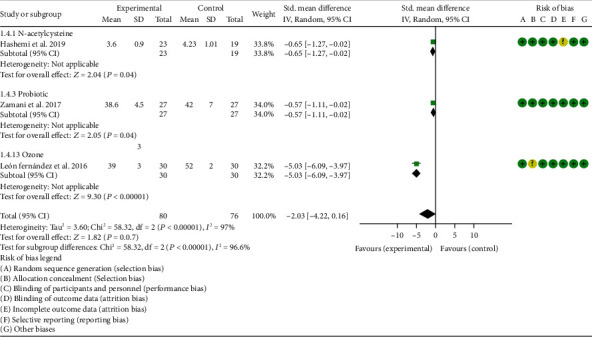
NO.

**Figure 8:**
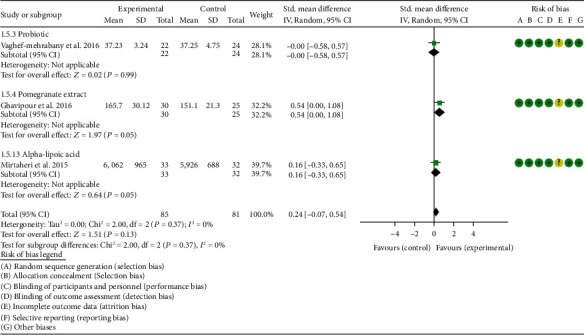
GPx.

**Figure 9:**
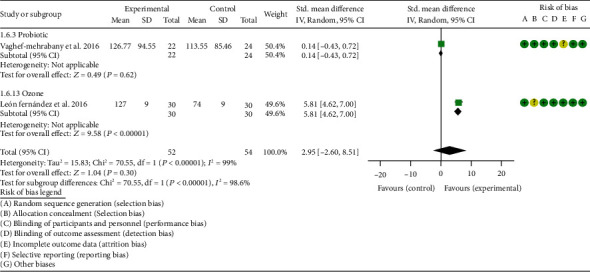
CAT.

**Figure 10:**
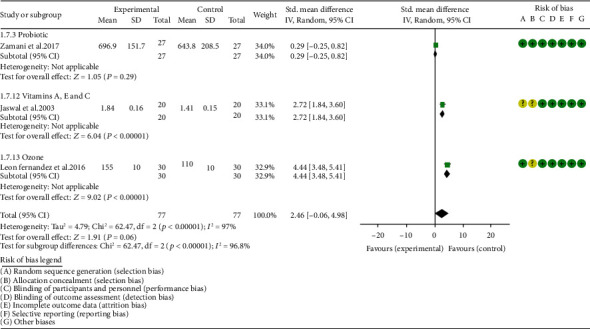
GSH.

1. *Oxidative Stress Index and Adverse Events*. A total of 11 RCTs reported MDA; the summary results showed that the MDA in the experiment group is lower (SMD -0.82, 95% CI -1.35 to -0.27, *P* = 0.003; random effect model) (Figure 4). Eight RCTs reported TAC; the summary results showed that the difference was of no statistical significance (SMD 0.27, 95% CI -0.21 to 0.75, *P* = 0.27; random effect model) (Figure 5). Four RCTs reported SOD; the summary results showed that the difference was of no statistical significance (SMD 0.12, 95% CI -0.16 to 0.40, *P* = 0.41; random effect model) (Figure 6). Three RCTs reported NO; the summary results showed that the difference was of no statistical significance (SMD -2.03, 95% CI -4.22 to 0.16, *P* = 0.07; random effect model) (Figure 7). Three RCTs reported GPx; the summary results showed that the difference was of no statistical significance (SMD 0.24, 95% CI -0.07 to 0.54, *P* = 0.13; random effect model) (Figure 8). Two RCTs reported CAT; the summary results showed that the difference was of no statistical significance (SMD 2.95, 95% CI -2.6 to 8.51, *P* = 0.30; random effect model) (Figure 9). Three RCTs reported GSH; the summary results showed that the difference was of no statistical significance (SMD 2.46, 95% CI -0.06 to 4.98, *P* = 0.06; random effect model) (Figure 10). Five RCTs reported adverse events; Abdollahzad et al. 2015 showed that no obvious adverse events were seen in the two groups. The summary results showed that the difference was of no statistical significance (RR 1.16, 95% CI 0.79 to 1.71, *P* = 0.45; random effect model).
2. *Clinical Efficacy Indexes*. Nine RCTs reported the number of swollen joints; the summary results showed that the number of swollen joints in the experiment group is lower (WMD -1.15, 95% CI -1.82 to -0.47, *P* = 0.0008; random effect model) (Figure 12). Seven RCTs reported the number of tender joints; the summary results showed that the number of tender joints in the experiments group is lower (WMD -2.50, 95% CI -3.12 to -1.89, *P* < 0.00001; random effect model) (Figure 13). Eleven RCTs reported the DAS28; the summary results showed that the DAS28 in the experiment group is lower (WMD -1.02, 95% CI -1.37 to -0.68, *P* < 0.00001; random effect model) (Figure 14). Nine RCTs reported the VAS; the summary results showed that the VAS in the experiment group is lower (SMD -0.66, 95% CI -1.02 to -0.31, *P* = 0.0003; random effect model) (Figure 15). Nine RCTs reported the HAQ; the summary results showed that the HAQ in the experiment group is lower (SMD -0.74, 95% CI -0.97 to -0.50, *P* < 0.00001; random effect model) (Figure 16).
3. *Inflammation Indexes*. Thirteen RCTs reported the ESR; the summary results showed that the ESR in the experiment group is lower (WMD -7.89, 95% CI -12.21 to -3.58, *P* = 0.0003; random effect model) (Figure 17). Eleven RCTs reported the CRP; the summary results showed that the CRP in the experiments group is lower (WMD -1.06, 95% CI -1.83 to -0.29, *P* = 0.007; random effect model) (Figure 18). Six RCTs reported the TNF-*α*; the summary results showed that the TNF-*α* in the experiment group is lower (SMD -0.49, 95% CI -0.89 to -0.09, *P* = 0.02; random effect model) (Figure 19). Four RCTs reported IL6; the summary results showed that the difference was of no statistical significance (SMD -0.32, 95% CI -1.28 to 0.63, *P* = 0.51; random effect model) (Figure 20).

#### 4.5.1. N-acetylcysteine

Three RCTs utilized to N-acetylcysteine treat RA. Hashemi et al. 2019 assessed the CRP, ESR, TNF-*α*, IL6, MDA, TAC, and NO. Batooei et al. 2018 assessed the DAS28, ESR, number of tender joints, number of swollen joints, HAQ, VAS, and adverse events. Yin et al. 2017 did not report any outcomes related to oxidative stress. The summary results of ESR showed that there was no statistically significant difference between the two groups after N-acetylcysteine intervention (WMD -0.87, 95% CI -2.85 to 1.12, *P* = 0.39) (Figure 17).

Hashemi et al. 2019 showed that the MDA and NO in the experiment group were lower (MDA (SMD -0.75, 95% CI -1.38 to -0.12, *P* = 0.02); NO (SMD -0.65, 95% CI -1.27 to -0.02, *P* = 0.04)) (Figures 4 and 7), while the IL6 in the experimental group was higher (SMD -0.05, 95% CI -0.66 to 0.56, *P* = 0.01) (Figure 20). The TAC, CRP, and TNF-*α* in Hashemi et al. 2019 between two groups were of no statistical significance (TAC (SMD -0.05, 95% CI -0.66 to 0.56, *P* = 0.87), CRP (WMD -0.20, 95% CI -0.91 to 0.51, *P* = 0.58), and TNF-*α* (SMD -0.28, 95% CI -0.89 to 0.33, *P* = 0.37)) (Figures 5, 18, and 19).

**Figure 11:**
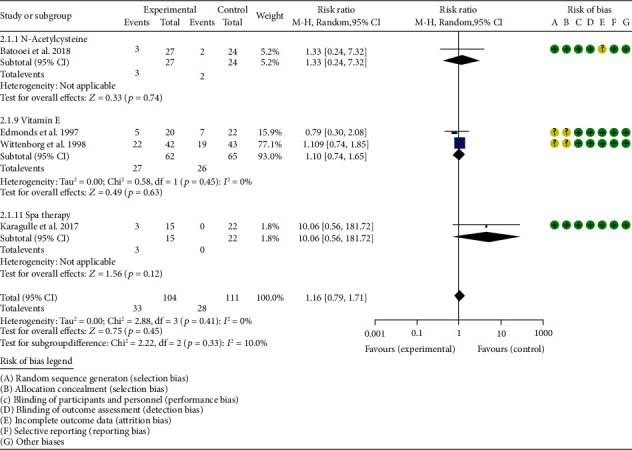
Adverse events.

**Figure 12:**
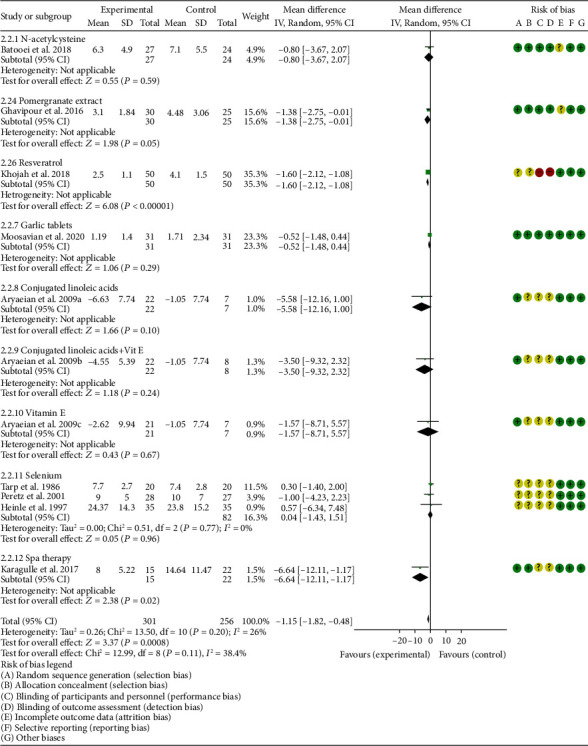
Number of swollen joints.

**Figure 13:**
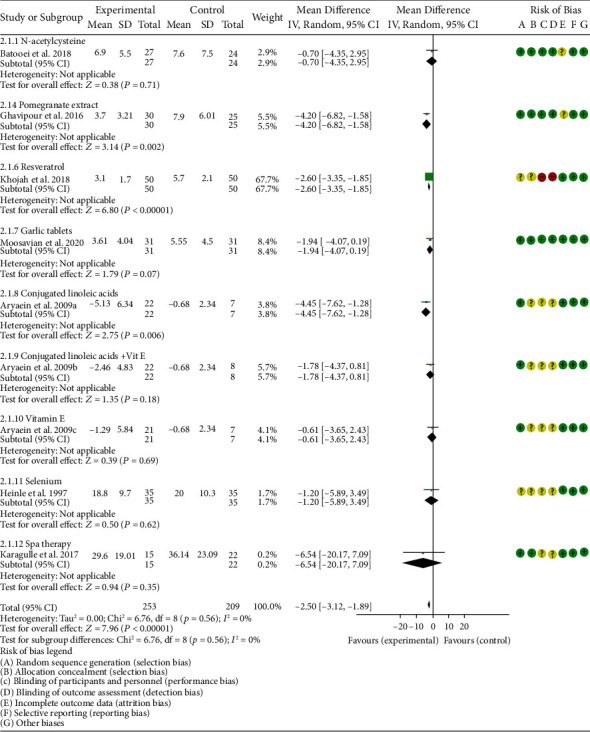
Number of tender joints.

**Figure 14:**
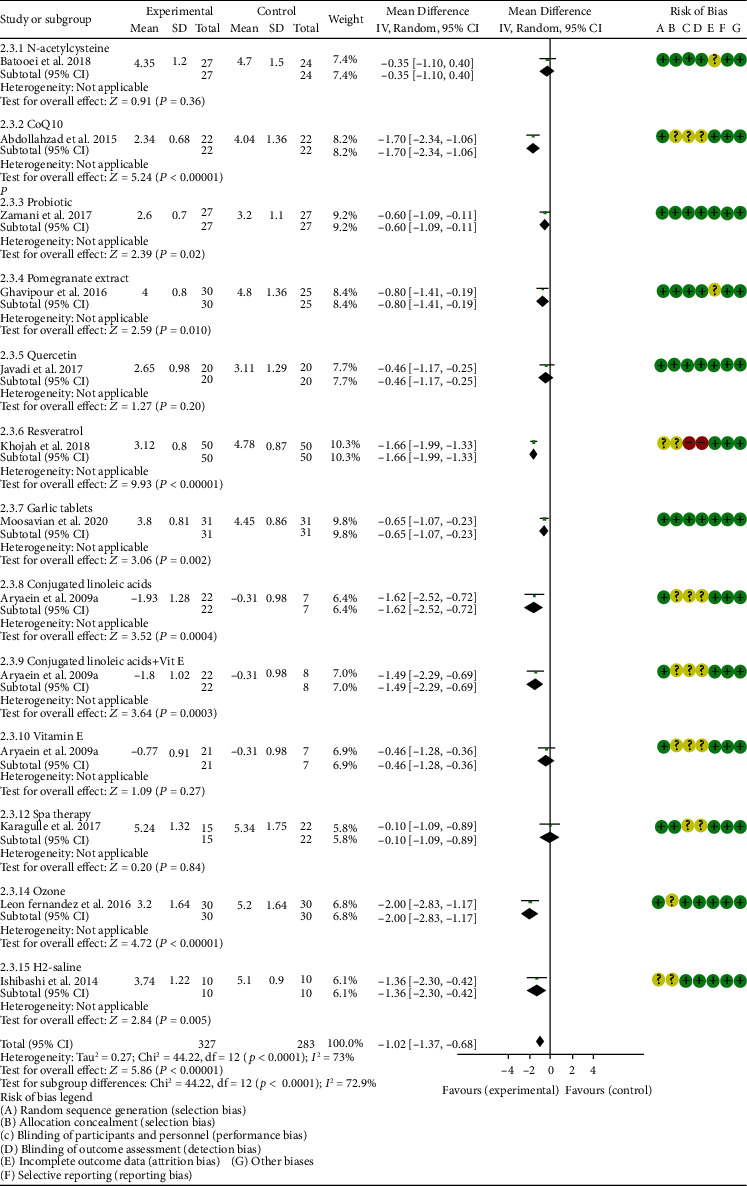
DAS28.

**Figure 15:**
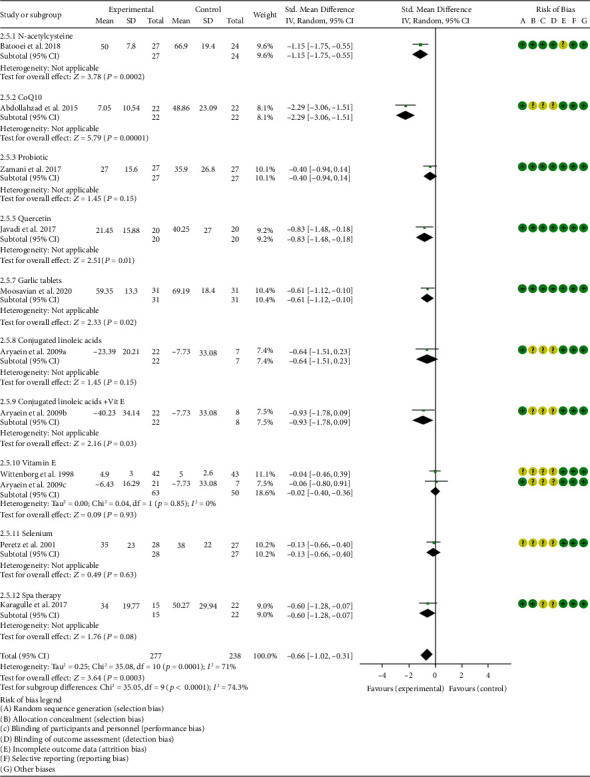
VAS.

**Figure 16:**
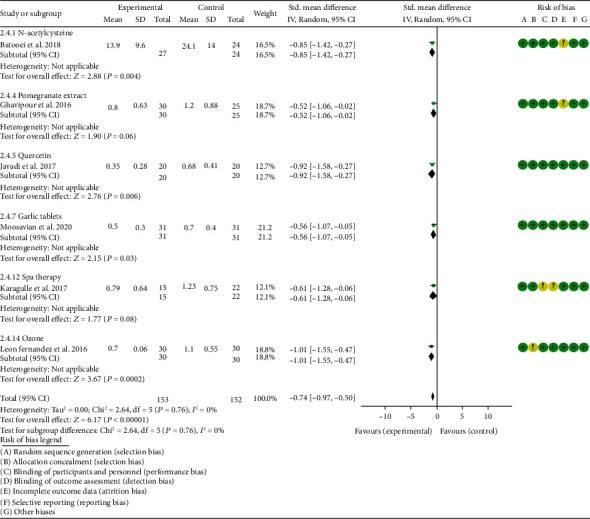
HAQ.

Batooei et al. showed that the adverse events, number of tender joints, number of swollen joints, and DAS28 between two groups were of no statistical significance (adverse events (RR 1.33, 95% CI 0.24 to 7.32, *P* = 0.74), number of swollen joints (WMD -0.80, 95% CI -3.67 to 2.07, *P* = 0.59), number of tender joints (WMD -0.70, 95% CI -4.35 to 2.95, *P* = 0.71), and DAS28 (WMD -0.35, 95% CI -1.10 to 0.40, *P* = 0.36)) (Figures 11–16). The HAQ and VAS in Batooei et al. were lower (VAS (SMD -1.15, 95% CI -1.75 to -0.55, *P* = 0.0002); HAQ (SMD -0.85, 95% CI -1.42 to -0.27, *P* = 0.004)) (Figures 18 and 19).

Abdollahzad et al. 2015 reported the effect of N-acetylcysteine combined with pulmonary rehabilitation exercise treatment on lung function in patients with RA-related interstitial lung disease; they found that N-acetylcysteine combined with pulmonary rehabilitation exercise therapy has a significant effect.

#### 4.5.2. Coenzyme Q10

**Figure 17:**
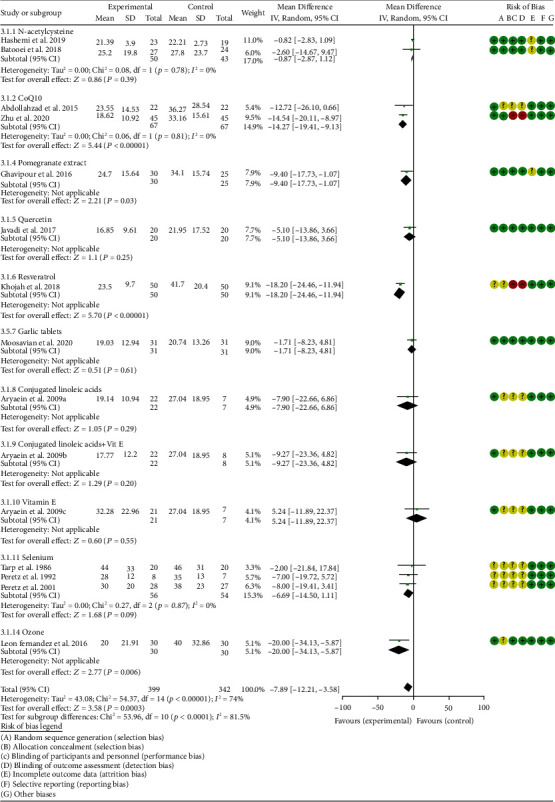
ESR.

Three RCTs utilized coenzyme Q10 to treat RA. Abdollahzad et al. 2015 assessed the MDA, TAC, DAS28, number of tender joints, number of swollen joints, ESR, TNF-*α*, IL6, VAS, and adverse events. Zhu et al. 2020 assessed the MDA, TAC, CRP, ESR, TNF-*α*, and IL6. The summary results in the CoQ10 subgroup showed that the MDA and ESR in CoQ10 groups were lower (MDA (SMD -0.71, 95% CI -1.06 to -0.36, *P* < 0.0001); ESR (WMD -14.27, 95% CI -19.41 to -9.13, *P* < 0.00001)) (Figures 4 and 17), while the difference of TAC between two groups was of no statistical significance (SMD -0.19, 95% CI -0.53 to 0.15, *P* = 0.43) (Figure 5). For TNF-*α* and IL6, the data representation of Abdollahzad et al. 2015 is median (interquartile range); hence, it cannot be merged with Zhu et al. 2020. However, both groups showed that after CoQ10 intervention, compared with the control group, the TNF-*α* in the experimental group decreased (*P* < 0.05). Meanwhile, Zhu et al. 2020 showed that compared with the control group, the IL6 in the experimental group decreased (*P* < 0.01) (Figure 20), while Abdollahzad et al. 2015 showed that there was no statistical difference between the two groups (*P* > 0.05).

Abdollahzad et al. 2015 showed that the DAS28 and VAS in experiments group were lower (DAS28 (WMD -1.70, 95% CI -2.34 to -1.06, *P* < 0.00001); VAS (SMD -2.29, 95% CI -3.06 to -1.51, *P* < 0.00001)) (Figures 14 and 15). It also showed that no obvious adverse events were seen in the two groups. Zhu et al. 2020 showed that the CRP in the experiment group was lower (WMD -3.92, 95% CI -6.51 to 1.33, *P* = 0.003). The data representation of the number of swollen joints and number of tender joints in Abdollahzad et al. 2015 is median (interquartile range), and the results showed that compared with the control group, the number of swollen joints and number of tender joints in the experimental group decreased.

#### 4.5.3. Probiotics

Two RCTs utilized probiotics to treat RA. Vaghef-Mehrabany et al. 2016 assessed the MDA, SOD, GPx, CAT, and TAC. Zamani et al. 2017 assessed the TAC, GSH, MDA, CRP, DAS28, and VAS. The summary results in the probiotic subgroup showed that the MDA in the probiotic groups was lower (SMD -0.71, 95% CI -1.06 to -0.36, *P* < 0.001) (Figure 4), while the difference of TAC between two groups was of no statistical significance (SMD -0.19, 95% CI -0.53 to 0.15, *P* = 0.27) (Figure 5).

Vaghef-Mehrabany et al. 2016 showed that the difference of SOD, GPx, and CAT between two groups was of no statistical significance (SOD (SMD -0.10, 95% CI -0.68 to 0.48, *P* = 0.73), GPx (SMD -0.00, 95% CI -0.58 to 0.57, *P* = 0.99), and CAT (SMD -0.14, 95% CI -0.43 to 0.72, *P* = 0.62)) (Figures 6, 8, and 9).

**Figure 18:**
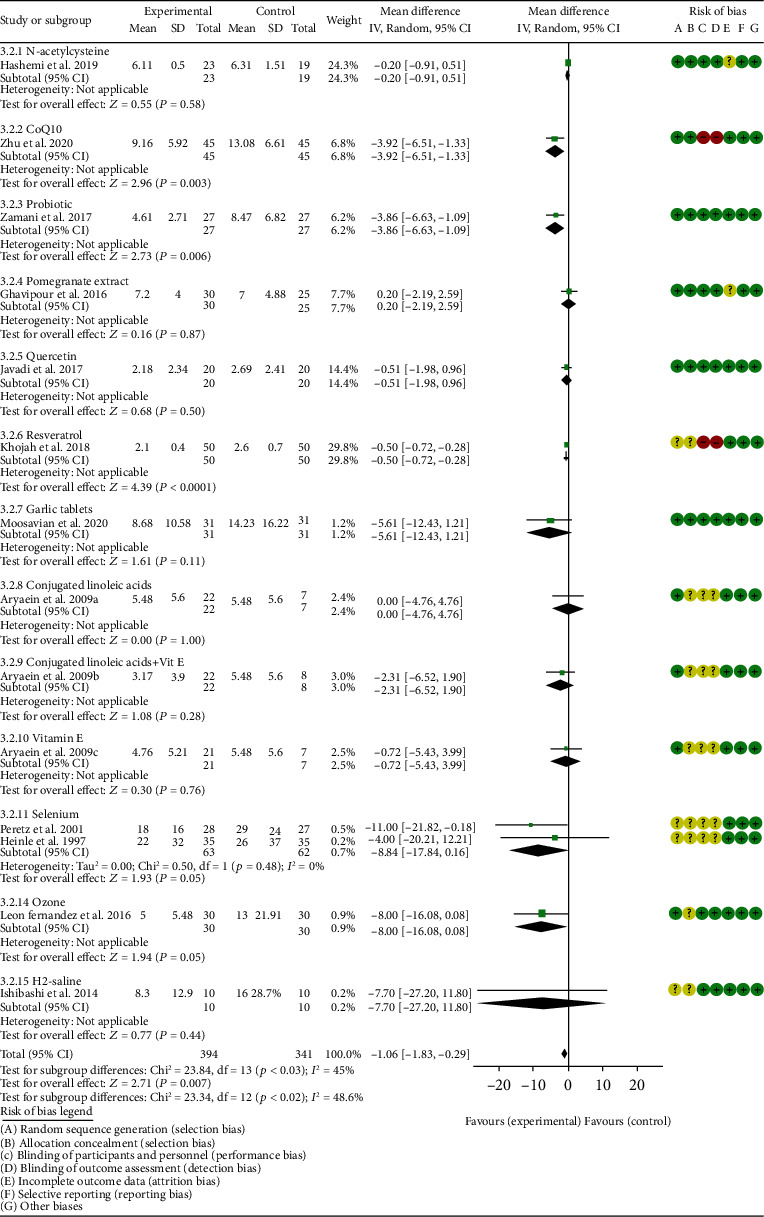
CRP.

Zamani et al. 2017 showed that the difference of GSH and VAS between two groups was of no statistical significance (GSH (SMD 0.29, 95% CI -0.20 to 0.78, *P* = 0.25); VAS (SMD -0.40, 95% CI –0.94 to 0.14, *P* = 0.15)) (Figures 10 and 15). It also showed that after probiotic intervention, compared with the control group, the DAS28 and CRP in the experimental group decreased (DAS28 (WMD -0.60, 95% CI -1.09 to -0.11, *P* = 0.02); CRP (WMD -3.86, 95% CI -6.63 to -1.09, *P* = 0.006)) (Figures 14 and 18).

#### 4.5.4. Pomegranate Extract

Only one RCT utilized pomegranate extract to treat RA. Ghavipour et al. 2016 assessed the DAS28, HAQ, ESR, CRP, number of tender joints, number of swollen joints, MDA, and GPx. The summary results in the pomegranate extract subgroup showed that the MDA in the pomegranate extract groups was higher (SMD 0.56, 95% CI 0.02 to 1.10, *P* = 0.04) (Figure 4), while the difference of GPx, HAQ, and CRP between two groups was of no statistical significance (GPx (SMD 0.54, 95% CI 0.00 to 1.08, *P* = 0.05), HAQ (SMD -0.52, 95% CI -1.06 to 0.02, *P* = 0.06), and CRP (WMD 0.20, 95% CI -2.19 to 2.59, *P* = 0.87)) (Figures 8, 16, and 18). It also showed that the number of swollen joints, number of tender joints, DAS28, and ESR were lower (number of swollen joints (WMD -1.38, 95% CI -3.67 to –0.01, *P* = 0.05), number of tender joints (WMD -4.20, 95% CI -6.82 to -1.58, *P* = 0.002), DAS28 (WMD -0.80, 95% CI -1.41 to -0.19, *P* = 0.010), and ESR (WMD -9.40, 95% CI -17.73 to -1.07, *P* = 0.003)) (Figures 12–14 and 17).

#### 4.5.5. Quercetin

Two RCTs utilized quercetin to treat RA. Javadi et al. 2017 assessed the DAS28, HAQ, ESR, CRP, TNF-*α*, number of tender joints, number of swollen joints, VAS, MDA, and TAC. Bae et al. 2009 reported CRP, TNF-*α*, and IL6.

Javadi et al. 2017 showed that MDA, VAS, and HAQ in the quercetin groups were lower (MDA (SMD -0.89, 95% CI -1.54 to -0.24, *P* = 0.008), VAS (SMD -0.83, 95% CI -1.48 to -0.18, *P* = 0.01), and HAQ (SMD -0. 92, 95% CI -1.58 to -0.27, *P* = 0.006)) (Figures 4, 15, and 16), while the difference of TAC, DAS28, ESR, and CRP between two groups was of no statistical significance (TAC (SMD -0.25, 95% CI -0.87 to 0.38, *P* = 0.44), DAS28 (WMD -0.46, 95% CI -1.17 to 0.25, *P* = 0.20), ESR (WMD -5.10, 95% CI –13.86 to 3.66, *P* = 0.25), and CRP (WMD -0.51, 95% CI -1.98 to 0.96, *P* = 0.50)) (Figures 5, 14, 17, and 18). The data representation of the TNF-*α*, number of tender joints, and number of swollen joints in Javadi et al. 2017 is median (interquartile range), and the results showed that compared with the control group, the TNF-*α* in the experimental group decreased (*P* < 0.05); meanwhile, the difference of the number of tender joints and number of swollen joints between the experimental group and the placebo group was of no statistical significance (*P* > 0.05).

**Figure 19:**
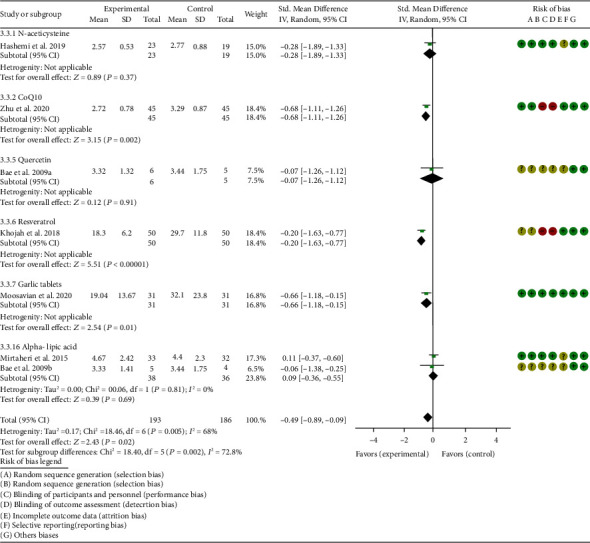
TNF-*α*.

**Figure 20:**
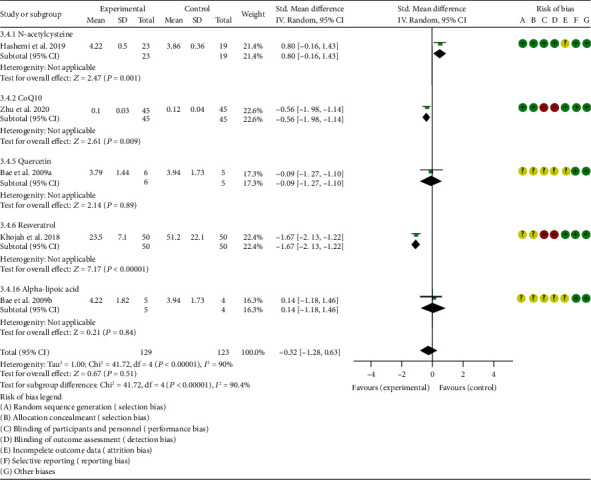
IL6.

Bae et al. 2009 showed that the difference of TNF-*α* and IL6 between two groups was of no statistical significance (TNF-*α* (SMD -0.07, 95% CI -1.26 to 1.12, *P* = 0.91); IL6 (SMD -0.09, 95% CI -1.27 to 1.10, *P* = 0.89)) (Figures 19 and 20). The data representation of the CRP is median (interquartile range), and the results showed that the difference of CRP between the experimental group and the placebo group was of no statistical significance (*P* > 0.05).

#### 4.5.6. Resveratrol

Only one RCT utilized resveratrol to treat RA, and it reported number of tender joints, number of swollen joints, DAS28, CRP, ESR, TNF-*α*, and IL6. The RCT evaluated 100 patients with RA. The control group used traditional RA therapy, while the test group was treated with 1 g resveratrol on the basis of traditional therapy. The treatment lasted 3 months. The study showed that the number of swollen and tender joints and the DAS28 in the resveratrol group were significantly reduced (*P* < 0.05) (Figures 12–14), and CRP, ESR, TNF-*α*, and IL6 were also reduced (*P* < 0.05) (Figures 17–20).

#### 4.5.7. Garlic Tablets

Only one RCT utilized garlic tablets to treat RA. Moosavian et al. 2020 assessed the HAQ, VAS, CRP, ESR, TNF-*α*, number of tender joints, number of swollen joints, MDA, and TAC. The summary results showed that the MDA in the experiment groups was lower (SMD -0.62, 95% CI -1.13 to -0.11, *P* = 0.008) (Figure 4), while the TAC in the experiment groups was higher (SMD 2.01, 95% CI 1.39 to 2.63, *P* < 0.00001) (Figure 5). It also showed that the difference of number of tender and swollen joints, ESR, and CRP between two groups was of no statistical significance (*P* > 0.05) (Figures 12, 13, 17, and 18), while the HAQ, VAS, and TNF-*α* in the experimental group were lower (*P* < 0.05) (Figures 15, 16, and 19).

#### 4.5.8. Vitamin E and Conjugated Linoleic Acids

Three RCTs utilized vitamin E to treat RA. Edmonds et al. 1997 reported adverse events; Wittenborg et al. 1998 reported VAS and adverse events; Aryaeian et al. 2009 reported VAS, ESR, CRP, DAS28, number of tender joints, and number of swollen joints. The summary results showed that the difference of adverse events and VAS between two groups was of no statistical significance (adverse events (RR 1.10, 95% CI 0.74 to 1.65, *P* = 0.64); VAS (SMD -0.02, 95% CI -0.04 to 0.36, *P* = 0.93)) (Figures 11 and 15).

Aryaeian et al. 2009 uses vitamin E alone and in combination with conjugated linoleic acids to intervene in RA patients. It showed that when conjugated linoleic acids were used alone, number of tender joints, number of swollen joints, and DAS28 were improved (*P* < 0.05) (Figures 12–14), but VAS, ESR, and CRP were not significantly improved (*P* > 0.05) (Figures 15, 17, and 18). When conjugated linoleic acids were combined with vitamin E, number of swollen joints, VAS, and DAS28 were improved (*P* < 0.05) (Figures 13–15), but number of tender joints, ESR, and CRP were not significantly improved (*P* > 0.05) (Figures 13, 17, and 18).

#### 4.5.9. Selenium

Four RCTs utilized selenium to treat RA. Tarp et al. 1986 reported the number of swollen joints and ESR; Peretz et al. 1992 reported VAS and ESR; Peretz et al. 2001 reported number of swollen joints, CRP, ESR, and VAS; Heinle et al. 1997 reported number of tender joints, number of swollen joints, and CRP. The summary results showed that the difference of number of swollen joints, ESR, and CRP between the two groups was of no statistical significance (number of swollen joints (WMD 0.04, 95% CI -1.43 to 1.51, *P* = 0.96), ESR (WMD -6.69, 95% CI -14.50 to 1.11, *P* = 0.09), and CRP (WMD -8.84, 95% CI -17.84 to 0.16, *P* = 0.05)) (Figures 12, 17, and 18). The data representation of the VAS in Peretz et al. 1992 is median (interquartile range), and the results showed that compared with the control group, the VAS in the experimental group decreased (*P* < 0.05). However, the difference of VAS in Peretz et al. 2001 between two groups was of no statistical significance (*P* > 0.05) (Figure 15). Heinle et al. 1997 also showed that the difference of number of tender joints between two groups was of no statistical significance (*P* > 0.05) (Figure 13).

#### 4.5.10. Spa Therapy

Only one RCT utilized spa therapy to treat RA. Karagülle et al. 2017 assessed the VAS, HAQ, DAS28, number of tender joints, number of swollen joints, MDA, SOD, and adverse events. The summary results showed that the difference of MDA, SOD, and adverse events between two groups was of no statistical significance (MDA (SMD 0.44, 95% CI -0.22 to 1.11, *P* = 0.19), SOD (SMD 0.28, 95% CI -0.08 to 0.95, *P* = 0.10), and adverse events (RR 1.16, 95% CI 0.79 to 1.71, *P* = 0.45)) (Figures 4, 5, and 11). It also showed the number of swollen joints (*P* < 0.05) (Figure 12), while the difference of number of tender joints, DAS28, VAS, and HAQ between two groups was of no statistical significance (*P* > 0.05) (Figures 13–16).

#### 4.5.11. Vitamins A, E, and C Combination

Only one RCT utilized vitamins A, E, and C combination to treat RA. Jaswal et al. 2003 assessed the MDA and GSH. The summary results showed that the MDA in the experiment group was lower (SMD -3.67, 95% CI -4.71 to -2.62, *P* < 0.00001) (Figure 4), while the GSH in the experiment group was higher (SMD 2.72, 95% CI 1.84 to 3.60, *P* < 0.00001) (Figure 10).

#### 4.5.12. Ozone

Only one RCT utilized ozone to treat RA. León Fernández et al. 2016 assessed the DAS28, HAQ, CRP, ESR, MDA, NO, GSH, SOD, and CAT. The summary results showed that the MDA, NO, DAS28, HAQ, and ESR in the experiment group were lower (SOD (SMD -2.47, 95% CI -4.71 to -2.62, *P* < 0.00001), NO (SMD -5.03, 95% CI -6.09 to -3.97, *P* < 0.00001), DAS28 (WMD -2.00, 95% CI -2.83 to -1.17, *P* < 0.00001), HAQ (SMD -1.01, 95% CI -1.55 to -0.47, *P* = 0.0002), and ESR (WMD -20.00, 95% CI -34.13 to -5.87, *P* = 0.006)) (Figures 4, 7, 14, 16, and 17), while the GSH and CAT in the experiment group were higher (GSH (SMD 4.44, 95% CI 3.48 to 5.41, *P* < 0.00001); CAT (SMD 5.81, 95% CI 4.62 to 7.00, *P* < 0.00001)) (Figures 9 and 10). The difference of SOD and CRP was of no statistical significance (SOD (SMD 0.44, 95% CI -0.08 to 0.95, *P* = 0.10); CRP (WMD -8.00, 95% CI -16.08 to 0.08, *P* = 0.05)) (Figures 6 and 18).

#### 4.5.13. H_2_-Saline

Only one RCT utilized H_2_-saline to treat RA. Ishibashi et al. 2014 reported DAS28, CRP, TNF-*α*, and IL6. Their study found that H_2_-saline may improve the clinical symptoms of RA patients (decreased DAS28) (*P* < 0.05) (Figure 14), while it has no obvious improvement effect on CRP (*P* > 0.05) (Figure 18). The indicators of TNF-*α* and IL6 could not be extracted, but the author reported that there was no significant change between the two compared with the placebo group (*P* > 0.05).

#### 4.5.14. Alpha-Lipoic Acid

Two RCTs utilized alpha-lipoic acid to treat RA. Mirtaheri et al. 2015 reported SOD, TAC, GPx, TNF-*α*, IL6, and CRP; Bae et al. 2009 reported CRP, TNF-*α*, and IL6. The summary results showed that the difference of TNF-*α* between two groups was of no statistical significance (SMD 0.09, 95% CI -0.36 to 0.55, *P* = 0.69). The data representation of the CRP in Mirtaheri et al. 2015 and Bae et al. 2009 is median (interquartile range), and both two RCTs showed that the results showed that the difference of CRP between the experimental group and the control group was of no statistical significance (*P* > 0.05). The data representation of the IL6 in Mirtaheri et al. 2015 is also median (interquartile range), but both two RCTs reported that the difference of IL6 between the experimental group and the control group was of no statistical significance (*P* > 0.05) (Figure 20). Mirtaheri et al. 2015 also showed that the difference of SOD, TAC, and GPx between two groups was of no statistical significance (TAC (SMD 0.44, 95% CI -0.06 to 0.93, *P* = 0.08), SOD (SMD -0.11, 95% CI -0.59 to 0.38, *P* = 0.66), and GPx (SMD 0.16, 95% CI -0.33 to 0.65, *P* = 0.52)) (Figures 5, 6, and 8).

### 4.6. Publication Bias of Outcomes

**Figure 21:**
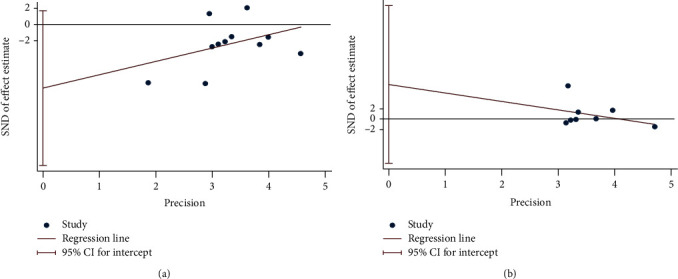
Publication bias of oxidative stress index: (a) MDA; (b) TAC.

**Figure 22:**
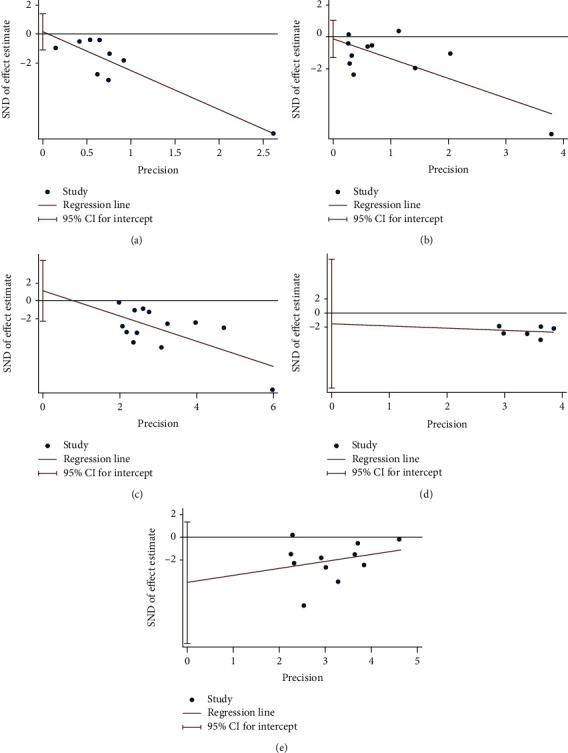
Publication bias of clinical efficacy indexes: (a) number of tender joints; (b) number of swollen joints; (c) DAS28; (d) HAQ; (e) VAS.

**Figure 23:**
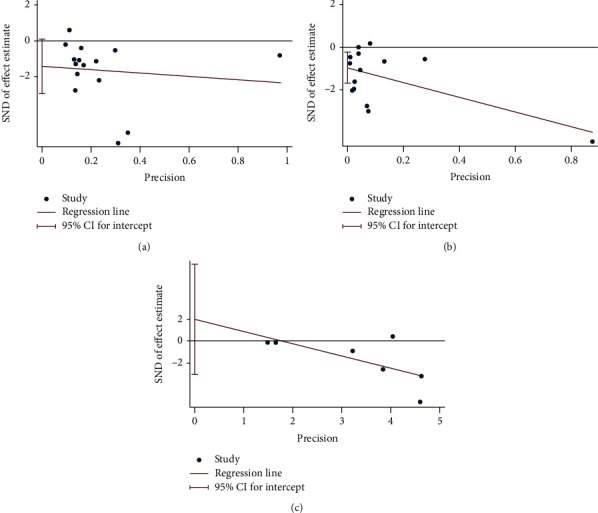
Publication bias of inflammation indexes: (a) ESR; (b) CRP; (c) TNF-*α*.

Finally, there are 10 outcomes with more than 5 RCTs: MDA, TAC, number of tender joints, number of swollen joints, DAS28, VAS, HAQ, ESR, CRP, and TNF-*α*. (1) For the oxidative stress index, the publication bias detection showed that the RCTs included in MDA may have publication bias (*P* = 0.094) (Figure 21(a)), while the that in TAC may not have publication bias (*P* = 0.329) (Figure 21(b)). (2) For clinical efficacy indexes, the publication bias detection showed that the RCTs may have publication bias (number of tender joints: *P* = 0.793, number of swollen joints: *P* = 0.791, DAS28: *P* = 0.476, HAQ: *P* = 0.66, and VAS: *P* = 0.126) (Figure 22). (3) For inflammation indexes, the publication bias detection showed that the RCTs included in ESR and CRP may have publication bias (ESR: *P* = 0.064; CRP: *P* = 0.014) (Figures 23(a) and 23(b)), while that in TNF-*α* may not have publication bias (*P* = 0.351) (Figure 23(b)).

## 5. Discussion

### 5.1. Summary of Main Outcomes

A total of 24 RCTs (28 records) and 1277 participants were included. The time span of RCTs is from 1986 to 2020. These RCTs involve 16 types of antioxidants or antioxidant therapies, and these therapies have varying degrees of improvement on oxidative stress in RA patients. (1) N-acetylcysteine: it may reduce the MDA and NO levels in RA patients, and the addition of N-acetylcysteine to conventional therapy will not increase the occurrence of adverse events. Meanwhile, it may relieve pain and improve the quality of life of patients (reduce VAS and HAQ). (2) Coenzyme Q10: it may reduce the MDA, ESR, and TNF-*α* in RA patients, and the addition of coenzyme Q10 to conventional therapy will not increase the occurrence of adverse events. Meanwhile, it may relieve pain and improve the patient's condition (reduce VAS and DAS28). Whether it can reduce IL6 is still inconclusive. (3) Probiotics: it may reduce the MDA and CRP levels and improve the patient's condition (reduce DAS28). It has not been observed to improve TAC, SOD, GPx, and CAT. (4) Pomegranate extract: interestingly, the MDA in pomegranate extract was higher, which is different from the results of other supplements. It has not been observed to improve GPx. Meanwhile, it may also reduce inflammation and relieve the condition (reduce number of swollen joints, number of tender joints, DAS28, and ESR). (5) Quercetin: it may reduce the MDA level in RA patients. Meanwhile, it may relieve pain and improve the quality of life of patients (reduce VAS and HAQ). (6) Resveratrol: the results showed that it may alleviate the patient's condition (reduce number of swollen and tender joints and the DAS28) and improve inflammation (reduce CRP, ESR, TNF-*α*, and IL6). (7) Garlic tablets: it may reduce the MDA level in RA patients and increase the TAC of RA patients. It may also relieve pain and improve the quality of life of patients (reduce VAS and HAQ) and reduce inflammation (reduce TNF-*α*). (8) Vitamin E and conjugated linoleic acids: whether conjugated linoleic acids were used alone (reduce the number of tender joints, number of swollen joints, and DAS28) or in combination with vitamin E (reduce number of swollen joints, VAS, and DAS28), it may improve the patient's condition. Meanwhile, the addition of vitamin E to conventional therapy will not increase the occurrence of adverse events. (9) Selenium: current research has not shown that selenium has a therapeutic effect on RA. What is interesting is that for VAS, RCT showed different results. Because the data is expressed in different ways, it cannot be combined, so it is impossible to draw a certain conclusion. (10) Spa therapy: it has no significant improvement on MDA and SOD, and it may reduce number of swollen joints. Meanwhile, spa therapy may not increase adverse events. (11) Vitamins A, E, and C combination: this combination may decrease MDA and increase GSH. (12) Ozone: it may reduce MDA and NO levels and increase CAT and GSH levels in RA patients. Meanwhile, it may also reduce inflammation and relieve the condition (reduce DAS28, HAQ, and ESR). (13) H_2_-saline: The H_2_-saline may improve the clinical symptoms of RA patients (decreased DAS28). (14) Alpha-lipoic acid: current research has not shown that alpha-lipoic acid has a therapeutic effect on RA.

In short, most antioxidants or antioxidant therapies can reduce MDA levels in RA patients, and a small number of therapies can increase GSH or TAC levels. And several antioxidants or antioxidant therapies may relieve pain and improve the quality of life of patients and the patient's condition. However, pomegranate extract may cause an increase in MDA. However, since there is only one RCT in most subgroups, the interpretation of the results still requires caution.

### 5.2. Possible Mechanism of Antioxidant Treatment of RA

In 1986, Koster et al. found that compared with healthy controls, the serum sulfhydryl concentration of RA patients was lower [47]. Considering that the sulfhydryl group may act as a scavenger of peroxides, this discovery had already indicated that the oxidative stress in RA patients was excessive. Subsequently, the characteristics of oxidative stress in the pathogenesis of RA have been reported successively [14, 48–51]. Oxidative stress is a state where the body's oxidation and antioxidant effects are out of balance and tend to be oxidized. Oxidative stress can cause inflammatory infiltration of neutrophils and promote the massive production of reactive oxygen species (ROS) and reactive nitrogen species (RNS) free radicals [13, 52]. ROS mainly includes superoxide anion (O_2_-) [53], hydrogen peroxide (H_2_O_2_) [54, 55], hypochlorous acid (HClO) [56], and hydroxyl radical (^·^OH) [57]. RNS mainly includes nitrogen monoxide (NO) [58–61] and peroxynitroso (ONOO-) [62, 63]. In addition, a variety of highly active molecules including oxidative stress will be produced under pathological conditions [56, 63, 64]. In addition to increasing the number of ROS/RNS under oxidative stress, antioxidants will also remove ROS/RNS substances or compounds, thereby inhibiting the oxidative stress process in cells [65]. Current research shows that there are mainly two different types of antioxidants, namely, enzymatic system and nonenzymatic system. The first type is mainly composed of SOD [65–67], CAT [68], GPx [69], glutathione reductase (GR) [70], and thioredoxin reductase [71]. ^·^O_2_- and H_2_O_2_ are the most ROS produced during oxidative stress [52, 69]. The former is cleared by SOD [65], and the latter is cleared by CAT [68], GPx [69], and perredoxin (PRX) [72]. The nonenzymatic antioxidant system is mainly composed of vitamins (A, C, and E), beta carotene, antioxidants, and minerals such as copper, ferritin, zinc, manganese, and selenium [52, 73].

Current basic research shows that oxidative stress plays a key role in the initiation and maintenance of systemic inflammation in RA [32, 45, 74, 75]. Under the pathological conditions of RA, ROS and RNS are produced by neutrophils, monocytes, and macrophages in joint tissues [76]. They can damage different types of cell structures in joints, including DNA, carbohydrates, proteins, and lipids [14, 17, 43, 74], leading to an imbalance of oxidative stress in joint tissues. Among them, the most common oxidation promoting factor (ROS/RNS) in RA joints is composed of ^·^O_2_-, H_2_O_2_, ^·^OH, NO^·^, ONOO-, HOCl, and LOO^·^ [32, 45, 74, 75]. In addition, in the occurrence and progression of RA joint damage, the oxidative stress imbalance and the inflammatory biological network are interconnected in multiple directions, which eventually leads to RA (synovitis) and forms a vicious circle. For example, ROS increases in RA patients [10] (mainly H_2_O_2_), which in turn activates the NF-*κ*B pathway [77]. NF-*κ*B signal transduction immunity promotes more IL-1 and TNF-*α*. Activated macrophages and T cells in the synovium may induce the production of ROS through the release of TNF and IL-1. This way further amplifies the inflammation of synovitis, forming a positive feedback, and worsening the process of RA synovitis [78, 79]. It is specifically manifested in the disease progression of RA patients. Compared with inactive RA patients, RA patients with active disease show higher ROS levels, more severe inflammatory factor levels, and lower antioxidant potential. Moreover, compared with healthy controls, these active RA patients have worse antioxidant capacity [74]. It is manifested by a higher degree of lipid peroxidation found in the synovial fluid and blood samples of these patients with possible RA [80, 81].

In addition, the increase in intra-articular pressure caused by chronic long-term inflammation in the joints of RA patients may lead to chronic hypoxia, which in turn increases the production of ROS in the joints of RA individuals [82]. The oxidation of type II collagen in the joints of patients with RA [10] and the increased production of matrix metalloproteinases [33] will cause oxidative damage to the matrix (extracellular environment) of the joints [10]. These oxidative stress factors can also induce stromal cells and joint cells (chondrocytes) to undergo programmed cell death caused by endoplasmic reticulum oxidative stress, which in turn leads to early joint damage in RA [10]. Further studies have also shown that oxidative stress can also cause other complications in RA patients. For example, high levels of inflammation and oxidative stress in RA patients can cause endothelial dysfunction and cause vascular damage to the circulatory system [83, 84]. Controlling the oxidative stress imbalance and inflammation in the preclinical and chronic stages of RA can avoid complications in the circulatory system of RA patients [84]. Aiming at the mechanism of oxidative stress in the clinical diagnosis and treatment of RA patients, oxidative stress biomarkers have been used as relevant markers and protocols to assess the disease activity and prognosis of RA patients [50, 82]. For example, Quiñonez-Flores et al. [50] found that lipid peroxidation (through MDA level) can be used to detect disease activity in RA patients (disease activity score DAS28), which expands the potential applicability of oxidative biomarkers in the diagnosis and prognosis of RA patients.

### 5.3. Characteristic Analysis of Included Studies

A total of 24 RCTs were included in this study, with a time span from 1986 to 2020. These 24 RCTs used a total of 14 different therapies; they were N-acetylcysteine, CoQ10, probiotic, pomegranate extract, quercetin, resveratrol, garlic tablets, vitamin E and conjugated linoleic acids, selenium, spa therapy, vitamins A, E, and C, ozone, H_2_-saline, and alpha-lipoic acid. Hashemi et al. 2019 [16], Batooei et al. 2018 [17], Abdollahzad et al. 2015 [19, 20], Zhu et al. 2020 [30], Vaghef-Mehrabany et al. 2016 [31], Zamani et al. 2017 [32], Ghavipour et al. 2016 [33], Javadi et al. 2017 [24, 25], Moosavian et al. 2020 [26, 27], Aryaeian et al. 2009 [36], Karagülle et al. 2017 [43], and León Fernández et al. 2016 [45] described the random sequence generation methods. Hashemi et al. 2019 [16], Batooei et al. 2018 [17], Zhu et al. 2020 [30], Vaghef-Mehrabany et al. 2016 [31], Zamani et al. 2017 [32], Ghavipour et al. 2016 [33], Javadi et al. 2017 [24, 25], Moosavian et al. 2020 [26, 27], and Karagülle et al. 2017 [43] described allocation concealment methods. The other RCTs failed to described the random sequence generation methods and/or allocation concealment methods. Since the main outcome of this meta-analysis is an objective indicator, it is less affected by whether or not blinding is used. Hence, although only Hashemi et al. 2019 [16], Batooei et al. 2018 [17], Zamani et al. 2017 [32], and Moosavian et al. 2020 [26, 27] uses blinding, all RCTs are assessed as low risk of bias regarding blinding. However, the implementation of blinding methods is still very important. Hashemi et al. 2019 [16], Batooei et al. 2018 [17], Vaghef-Mehrabany et al. 2016 [31], Ghavipour et al. 2016 [33], and Bae et al. 2009 [34] have incomplete outcome data. In addition, 2 RCTs were from Belgium; 2 RCTs were from China; 2 RCTs were from Germany; 8 RCTs were from Iran; Bae et al. 2009 was from Korea; Khojah et al. 2018 was from Egypt; Edmonds et al. 1997 was from the UK; Tarp et al. 1986 was from Denmark; Karagülle et al. 2017 was from Turkey; Jaswal et al. 2003 was from India; León Fernández et al. 2016 was from Cuba; and Ishibashi et al. 2014 was from Japan. The included RCTs in this study showed that the included patients were mainly women. This is consistent with the facts: the incidence of RA is higher in women than in men, and women are 2 to 3 times that of men, and it occurs more frequently in 30-50 years of age [85–87]. Therefore, the results of this study mainly show the effect of antioxidant therapy in women with RA. Although it also shows potential effects for men, more samples are needed to further give better evidence. Most RCTs reported disease duration, baseline CRP, baseline ESR, and baseline DAS28, while a small number of RCTs did not report these baseline data. Baseline data suggest that the disease duration of most patients is more than 5 years, and most RCTs select moderate to severe patients in the active phase for the study.

In general, the quality of RCTs is medium to high. However, since most RCTs are not blinded, and a small number of studies have not conducted allocation concealment and description of random sequence generation methods, the interpretation of the results still needs to be cautious.

### 5.4. Strengths and Limitations of This Research and Inspiration for Future Research

The strengths of this research is that it is the first meta-analysis involving the improvement of oxidative stress in RA patients with antioxidants and antioxidant therapies. The RCTs collected in this study span 34 years (1986-2020) involving 1277 participants, and a comprehensive systematic review and meta-analysis of previous related studies have been extensively conducted. The quality of RCT is generally high. In addition, the RCTs included this time involve multiple countries and ethnic groups, including Belgium, China, Cuba, Denmark, Egypt, the UK, Germany, India, Iran, Japan, Korea, and Turkey, which makes the results more applicable.

The limitations of this research is that most subgroups have only one RCT (such as the N-acetylcysteine, pomegranate extract, quercetin, garlic tablets, spa therapy, vitamins A, E, and C combination, and ozone subgroup in MDA; all subgroups of SOD, NO, GPx, CAT, and GSH). This affects the credibility of the results, because only one RCT cannot represent all the population. Meanwhile, there are many RCTs that do not involve indicators of oxidative stress, such as Yin et al. 2017 [18], Bae et al. 2009 [34], Khojah et al. 2018 [35], Aryaeian et al. 2009 [36], Tarp et al. 1986 [39], Peretz et al. 1992 [40], Peretz et al. 2001 [41], Heinle et al. 1997 [42], and Ishibashi et al. 2014 [46]. Therefore, more research on the effects of these therapies on oxidative stress indicators in RA patients is needed. Meanwhile, the intervention duration of these RCTs is different, which may affect the effect of drug intervention in RA. In addition, although most RCTs are considered to be of high quality, blinding methods (such as Yin et al. 2017 [18], Abdollahzad et al. 2015 [19, 20], Zhu et al. 2020 [30], Vaghef-Mehrabany et al. 2016 [31], Ghavipour et al. 2016 [33], Javadi et al. 2017 [24, 25], Bae et al. 2009 [34], Khojah et al. 2018 [35], Aryaeian et al. 2009 [36], Edmonds et al. 1997 [37], Wittenborg et al. 1998 [38], Tarp et al. 1986 [39], Peretz et al. 1992 [40], Peretz et al. 2001 [41], Heinle et al. 1997 [42], Karagülle et al. 2017 [43], Jaswal et al. 2003 [44], León Fernández et al. 2016 [45], and Ishibashi et al. 2014 [46]) are not used. The main reason they were rated as low risk of bias was that the main outcome indicators were objective indicators (serum MDA, etc.). However, we still need to be vigilant, because the failure to implement blinding may affect other outcome indicators that are not focused on in this study. Therefore, in the future, more well-designed, randomized controlled double-blind clinical trials are needed to verify or modify the outcome indicators.

In MDA outcomes, there was a result contrary to most results: the MDA in the pomegranate extract group was higher than that of the control group. This is a very interesting result, because it suggests that pomegranate extract may have a reverse effect. However, since there is only one RCT, the result is unstable. Therefore, we look forward to more pomegranate extract-related RCTs in the future. In addition, although current RCTs show that antioxidants or antioxidant therapies do not increase the incidence of adverse events, most RCTs do not report safety outcomes. Therefore, it is expected that future RCTs will report more on the incidence of corresponding adverse events to determine the safety of those therapy.

## 6. Conclusion

Oxidative stress plays an important role in the pathophysiology of RA. This study showed through systematic reviews and meta-analysis that although there are currently fewer RCTs for antioxidant therapy, the existing evidence shows potential benefits, mainly in reducing MDA and increasing TAC and GSH. Meanwhile, it was also found that the combination of antioxidant therapy and conventional therapy is the main choice for reducing RA disease and preventing cardiovascular complications in the future. However, considering the small number of patients recruited, the study design varies greatly between different RCT studies, and the characteristics of RA participants included in different RCT studies are not the same; it is difficult to immediately extrapolate these results to general RA patients. In the future, more large samples and higher quality RCTs are needed to provide high-quality evidence, so as to provide more clinical reference information for the antioxidant treatment of RA.

## Figures and Tables

**Table 1:** The characteristics of the included studies.

Subgroup	Study	Trial registration number	Country	Sample size (female/male)		Intervention		Relevant outcomes	Mean age (years)		Disease duration (years)		Disease severity		Baseline CRP (mg/L)		Baseline ESR (mm/h)		Baseline DAS28		Duration
				Trial group	Control group	Trial group	Control group		Trial group	Control group	Trial group	Control group	Trial group	Control group	Trial group	Control group	Trial group	Control group	Trial group	Control group	
N-acetylcysteine	Hashemi et al. 2019 [16]	IRCT2015071722965N2	Iran	23 (20/3)	19 (19/0)	N-acetylcysteine 600 mg Bid+conventional treatment (mainly methotrexate, sulfasalazine, hydroxychloroquine, prednisolone, calcium D, folic acid, nonsteroidal anti-inflammatory drugs)	Placebo+conventional treatment (mainly methotrexate, sulfasalazine, hydroxychloroquine, prednisolone, calcium D, folic acid, nonsteroidal anti-inflammatory drugs)	CRP, ESR, TNF-*α*, IL6, MDA, TAC, NO	53.91 ± 13.90	50.68 ± 11.15	10.52 ± 7.19	10.05 ± 7.56	Moderate: 13 patients; severe: 10 patients	Moderate: 10 patients; severe: 9 patients	8.57 ± 0.99	7.89 ± 1.11	26.91 ± 4.29	26.26 ± 6.77	—	—	12 weeks
	Batooei et al. 2018 [17]	Cannot be found	Iran	27 (22/5)	24 (23/1)	N-acetylcysteine 600 mg Bid+conventional treatment (mainly methotrexate, sulfasalazine, hydroxychloroquine, calcium D, folic acid)	Placebo+conventional treatment (mainly methotrexate, sulfasalazine, hydroxychloroquine, calcium D, folic acid)	DAS28, ESR, number of tender joints, number of swollen joints, HAQ, VAS, adverse events	53.2 ± 12.5	51.6 ± 11.3	10 ± 7.0	9.8 ± 8.2	Moderate: 16 patients; severe: 11 patients	Moderate: 13 patients; severe: 11 patients	31.4 ± 19.6	29.2 ± 19.3	—	—	5.1 ± 1.2	5.3 ± 1.1	12 weeks
	Yin et al. 2017 [18]	—	China	48 (42/6)		N-acetylcysteine 600 mg+conventional treatment (mainly sulfasalazine, methotrexate)	Conventional treatment (mainly sulfasalazine, methotrexate)	None	36-75		—	—	—	—	—	—	—	—	—	—	12 weeks
CoQ10	Abdollahzad et al. 2015 [19, 20]	IRCT201311014105N16	Iran	22 (19/3)	22 (20/2)	CoQ10 100 mg+conventional treatment (mainly methotrexate, sulfasalazine, hydroxychloroquine, prednisolone)	Placebo (wheat starch)+conventional treatment (mainly methotrexate, sulfasalazine, hydroxychloroquine, prednisolone)	DAS28, number of tender joints, number of swollen joints, TNF-*α*, IL6, VAS, ESR, MDA, TAC, adverse events	48.77 ± 11.58	50.41 ± 11.28	6.91 ± 5.87	6.94 ± 6.50	All patients are moderate and severe		—	—	39.64 ± 25.53	40.27 ± 25.75	5.01 ± 1.21	4.88 ± 0.96	8 weeks
	Zhu et al. 2020 [30]	—	China	45 (35/10)	45 (37/8)	CoQ10 10 mg Tid+conventional treatment (mainly methotrexate, low-dose prednisone)	Placebo (wheat starch)+ conventional treatment (mainly methotrexate, low-dose prednisone)	CRP, ESR, TNF-*α*, IL6, MDA, TAC	48.15 ± 11.68	47.36 ± 12.11	5.2 (1.9, 8.6)^∗^	5.1 (2.2, 8.1)^∗^	—	—	56.15 ± 20.13	57.42 ± 18.46	42.21 ± 16.11	41.26 ± 15.26	—	—	12 weeks
Probiotic	Vaghef-Mehrabany et al. 2016 [31]	IRCT201207024105N10	Iran	22 (22/0)	24 (24/0)	Probiotic (Lactobacillus casei 01)+conventional treatment (mainly methotrexate, hydroxychloroquine, prednisolone)	Placebo+conventional treatment (mainly methotrexate, hydroxychloroquine, prednisolone)	MDA, SOD, GPx, CAT, TAC	41.14 ± 12.65	44.29 ± 9.77	4.75 (3.0, 9.0)^∗^	5.25 (3.75, 10.0)^∗^	All patients are inactive to moderate (DAS28 < 5.1)		—	—	—	—	<5.1		8 weeks
	Zamani et al. 2017 [32]	IRCT201611165623N94	Iran	27 (22/5)	27 (24/3)	Synbiotic capsule (Lactobacillus acidophilus, Lactobacillus casei, and Bifidobacterium bifidum (2 × 10^9^ colony‐forming units/g each) + 800 mg inulin)	Placebo (starch only)	CRP, DAS28, VAS, NO, TAC, GSH, MDA	49.3 ± 11.0	49.5 ± 12.9	7.7 ± 6.1	7.5 ± 6.4	All patients are moderate and severe		6.03 ± 4.84^#^	5.64 ± 5.14^#^	—	—	4.2 ± 0.7	3.5 ± 0.8	8 weeks
Pomegranate extract	Ghavipour et al. 2016 [33]	IRCT201202183236N2	Iran	30 (20/10)	30 (20/10)	Pomegranate extract (contained 40% ellagic acid) with no changes to current medication (mainly methotrexate, hydroxychloroquine, sulfasalazine, and prednisolone)	Placebo with no changes to current medication (mainly methotrexate, hydroxychloroquine, sulfasalazine, and prednisolone)	DAS28, HAQ, ESR, CRP, number of tender joints, number of swollen joints, MDA, GPx	48.4 ± 11.4	49.1 ± 12.2	10.9 ± 5.8	12.3 ± 5.8	Active RA		29.0 ± 15.6	30.6 ± 19.6	8.0 ± 4.2	6.6 ± 4.5	4.9 ± 0.8	4.7 ± 1.1	8 weeks
Quercetin	Javadi et al. 2017 [24, 25]	IRCT138807252394N2	Iran	20 (20/0)	20 (20/0)	Quercetin 500 mg+conventional treatment (mainly methotrexate, hydroxychloroquine, sulfasalazine, cyclosporine, prednisolone, NSAIDs)	Placebo+conventional treatment (mainly methotrexate, hydroxychloroquine, sulfasalazine, cyclosporine, prednisolone, NSAIDs)	DAS28, HAQ, ESR, CRP, TNF-*α*, number of tender joints, number of swollen joints, VAS, MDA, TAC	46.55 ± 9.94	48.00 ± 8.39	5.17 ± 3.83	4.87 ± 3.03	Mild to moderate disease activity		2.89 ± 2.95^#^	3.28 ± 2.32^#^	19.00 ± 8.62	21.10 ± 12.38	3.22 ± 0.93	3.13 ± 1.1	8 weeks
	Bae et al. 2009 [34]	—	Korea	20 (19/1)		Quercetin+vitamin C (166 mg 133 mg/capsule)+conventional treatment (mainly hydroxychloroquine, sulfasalazine, methotrexate with folate, bucillamine, NSAID, low-dose steroid)	Placebo+conventional treatment (mainly hydroxychloroquine, sulfasalazine, methotrexate with folate, bucillamine, NSAID, low-dose steroid)	CRP, TNF-*α*, IL6	52.1 ± 10.3		10.2 ± 5.9		Not known		0.85 (0.28, 4.00)^∗^	1.05 (0.22, 6.44)^∗^	—	—	—	—	4 weeks
Resveratrol	Khojah et al. 2018 [35]	—	Egypt	50 (36/14)	50 (32/18)	Resveratrol 1000 mg+conventional treatment	Placebo+conventional treatment	Number of tender joints, number of swollen joints, DAS28, CRP, ESR, TNF-*α*, IL6	46.5 ± 12.3	44.2 ± 16.4	9.4 ± 5.8	9.8 ± 5.5	Not known		2.7 ± 0.7	2.9 ± 0.8	39.4 ± 11.5	43.8 ± 14.8	4.62 ± 0.99	4.91 ± 0.92	12 weeks
Garlic tablets	Moosavian et al. 2020 [26, 27]	IRCT20141108019853N6	Iran	31 (31/0)	31 (31/0)	Garlic tablets 500 mg (equivalent to 2500 mg of fresh garlic and containing 2.5 mg allicin) Bid with no changes to current medication (mainly prednisolone, methotrexate, sulfasalazine)	Placebo with no changes to current medication (mainly prednisolone, methotrexate, sulfasalazine)	HAQ, VAS, CRP, ESR, TNF-*α*, number of tender joints, number of swollen joints, MDA, TAC	51.06 ± 13.8	51.39 ± 10.38	6.58 ± 7.75	6.61 ± 8.11	All patients are moderate and severe		13.44 ± 13.76	13.57 ± 14.04	23.63 ± 13.82	20.10 ± 11.74	4.61 ± 0.92	4.52 ± 0.78	8 weeks
Conjugated linoleic acids	Aryaeian et al. 2009 [36]	—	Iran	22 (19/3)	22 (19/3)	Conjugated linoleic acid 2 g with no changes to current medication (mainly hydroxychloroquine, choloroquine and methotrexate, lower amounts of NSAIDs)	Placebo with no changes to current medication (mainly hydroxychloroquine, choloroquine and methotrexate, lower amounts of NSAIDs)	VAS, ESR, CRP, DAS28, number of tender joints, number of swollen joints	46.23 ± 13.07	47.95 ± 11.14	9.95 ± 8.41	8.88 ± 8.65	Mile: 11; moderate: 8; severe: 3	Mile: 8; moderate: 13; severe: 1	7.19 ± 10.13	6.44 ± 7.90	26.81 ± 15.50	28.36 ± 21.55	4.63 ± 1.26	4.35 ± 0.95	12 weeks
Conjugated linoleic acids+vitamin E	Aryaeian et al. 2009 [36]	—	Iran	22 (17/5)		Conjugated linoleic acids 2 g+vitamin E 400 mg with no changes to current medication (mainly hydroxychloroquine, choloroquine and methotrexate, lower amounts of NSAIDs)			43.77 ± 12.75	47.95 ± 11.14	7.64 ± 6.19	8.88 ± 8.65	Mile: 8; moderate: 10; severe: 4	Mile: 8; moderate: 13; severe: 1	5.24 ± 6.44	6.44 ± 7.90	28.45 ± 17.26	28.36 ± 21.55	4.59 ± 1.11	4.35 ± 0.95	12 weeks
Vitamin E	Aryaeian et al. 2009 [36]	—	Iran	21 (17/4)		Vitamin E 400 mg with no changes to current medication (mainly hydroxychloroquine, choloroquine and methotrexate, lower amounts of NSAIDs)			49.33 ± 11.89	47.95 ± 11.14	7.24 ± 5.82	8.88 ± 8.65	Mile: 7; moderate: 12; severe: 2	Mile: 8; moderate: 13; severe: 1	9.06 ± 14.33	6.44 ± 7.90	40.43 ± 26.22	28.36 ± 21.55	4.52 ± 1.08	4.35 ± 0.95	12 weeks
	Edmonds et al. 1997 [37]	—	The UK	20 (16/4)	22 (15/7)	Vitamin E 600 mg Bid with no changes to current medication (mainly NSAID, methotrexate, Salazopyrin, azathioprine, D-penicillamine, Myocrisin, sulfasalazine, corticosteroids)	Placebo with no changes to current medication (mainly NSAID, methotrexate, Salazopyrin, azathioprine, D-penicillamine, Myocrisin, sulfasalazine, corticosteroids)	Adverse events	24-75	32-66	—	—	Not known		—	—	—	—	—	—	12 weeks
	Wittenborg et al. 1998 [38]	—	Germany	42 (39/3)	43 (30/13)	Vitamin E 400 mg Tid with no changes to basic treatment and physical therapy; other NSAIDs are not allowed during treatment	Diclofenac-sodium 50 mg Tid with no changes to basic treatment and physical therapy; other NSAIDs are not allowed during treatment	VAS, adverse events	61 ± 9	58 ± 9	10 ± 9	11 ± 11	Not known		—	—	—	—	—	—	3 weeks
Selenium	Tarp et al. 1986 [39]	—	Denmark	20 (14/6)	20 (15/5)	Selenium 256 *μ*g with no changes to current medication (mainly gold, 2 D-penicillamine, antimalarials, and NSAIDs)	Placebo with no changes to current medication (mainly gold, 2 D-penicillamine, antimalarials, and NSAIDs)	Number of swollen joints, ESR	54.3 ± 12.4	54.6 ± 12.7	16.4 ± 10.1	10.5 ± 8.0	Not known		—	—	47 ± 35	39 ± 26	—	—	24 weeks
	Peretz et al. 1992 [40]	—	Belgium	8 (8/0)	7 (7/0)	Selenium 200 *μ*g	Placebo	VAS, ESR	61 ± 11		—	—	Not known		—	—	23 ± 15	30 ± 17	—	—	24 weeks
	Peretz et al. 2001 [41]	—	Belgium	28 (21/7)	27 (20/7)	Selenium 200 *μ*g with stable dose of corticosteroids and of disease-modifying drugs (such as NSAIDs and low-dose glucocorticosteroids)	Placebo with stable dose of corticosteroids and of disease-modifying drugs (such as NSAIDs and low-dose glucocorticosteroids)	Number of swollen joints, CRP, ESR, VAS	61 ± 13	60 ± 13	—	—	All patients are moderate		28 ± 17	28 ± 17	34 ± 16	29 ± 12	—	—	12 weeks
	Heinle et al. 1997 [42]	—	Germany	38 (37/3)	32 (30/2)	Selenium 200 *μ*g	Placebo with no changes to basic treatment and the cortisone or NSAIDs were adjusted as needed	Number of tender joints, number of swollen joints, CRP	58.2 ± 12.78	57.2 ± 13.27	12.69 ± 8.3	12.03 ± 7.8	Not known		23.4 ± 18	17.1 ± 28	—	—	—	—	12 weeks
Spa therapy	Karagülle et al. 2017 [43]	—	Turkey	15 (13/2)	22 (22/0)	Spa therapy+standard drug treatment (mainly methotrexate, hydroxychloroquine, leflunomide, or sulfasalazine; glucocorticoids and NSAIDs)	Standard drug treatment (mainly methotrexate, hydroxychloroquine, leflunomide, or sulfasalazine; glucocorticoids and NSAIDs)	VAS, HAQ, DAS28, MDA, number of tender joints, number of swollen joints, SOD, adverse events	53.3 ± 11.1	52.3 ± 12.3	12.3 ± 12.9	13.4 ± 12.0	Not known		—	—	38.5 ± 18.0	38.5 ± 18.0	6.5 ± 0.9	5.9 ± 1.6	12 weeks
Vitamins A, E, and C	Jaswal et al. 2003 [44]	—	India	20 (not known)	20 (not known)	Vitamins A, E, and C+conventional treatment	Conventional treatment	MDA, GSH	—		—	—	Not known		—	—	—	—	—	—	12 weeks
Ozone	León Fernández et al. 2016 [45]	—	Cuba	30 (28/2)	30 (27/3)	Ozone+methotrexate 12.5 mg+Ibuprophen 400 mg+folic acid 5 mg	Methotrexate 12.5 mg+Ibuprophen 400 mg+folic acid 5 mg	DAS28, HAQ, CRP, ESR, MDA, NO, GSH, SOD, CAT	57 ± 7	53 ± 7	11 ± 3	7 ± 2	All patients are moderate		16 ± 4	21 ± 7	36 ± 6	40 ± 6	6.4 ± 0.2	5.6 ± 0.3	3 weeks
H_2_-saline	Ishibashi et al. 2014 [46]	—	Japan	12 (10/2)	12 (10/2)	H_2_-saline 500 ml	Placebo	DAS28, CRP, TNF-*α*, IL6	62.4 ± 18.4	68.2 ± 12.6	Mean: 4.25	Mean: 4.92	Not known		14.7 ± 17	13 ± 20	—	—	5.10 ± 0.96	5.18 ± 1.16	4 weeks
Alpha-lipoic acid	Mirtaheri et al. 2015 [28, 29]	IRCT201205263140N5	Iran	33 (33/0)	32 (32/0)	Alpha-lipoic acid 1200 mg with no changes to current medication (mainly prednisolone, methotrexate, hydroxychloroquine, sulfasalazine, calcium and vitamin D, folic acid)	Placebo (maltodextrin) with no changes to current medication (mainly prednisolone, methotrexate, hydroxychloroquine, sulfasalazine, calcium and vitamin D, folic acid)	SOD, TAC, GPx, TNF-*α*, IL6, CRP	36.09 ± 8.77	38.28 ± 8.63	7.26 ± 4.9	6.78 ± 4.72	All patients are inactive to moderate (DAS28 < 5.1)		3 (1.1, 10.1)^∗^^#^	3.5 (0.9, 9.5)^∗^^#^	—	—	2.1 ± 0.76	2.14 ± 0.72	8 weeks
	Bae et al. 2009 [34]	—	Korea	20 (19/1)		*α*-Lipoic acid (300 mg/capsule)+conventional treatment (mainly hydroxychloroquine, sulfasalazine, methotrexate with folate, bucillamine, NSAID, low-dose steroid)	Placebo+conventional treatment (mainly hydroxychloroquine, sulfasalazine, methotrexate with folate, bucillamine, NSAID, low-dose steroid)	CRP, TNF-*α*, IL6	52.1 ± 10.3		10.2 ± 5.9		Not known		0.84 (0.14, 4.28)^∗^	1.05 (0.22, 6.44)^∗^	—	—	—	—	4 weeks

## Data Availability

All data generated or analyzed during this study are included in this published article.

## Supplementary Materials

Supplementary 1 Table S1: search strategies for PubMed and Embase.

Supplementary 2 PRISMA 2020 checklist: checklist.
