# Supplementary material for: The Efficacy of Antioxidative Stress Therapy on Oxidative Stress Levels in Rheumatoid Arthritis: A Systematic Review and Meta-analysis of Randomized Controlled Trials
Source: Oxid Med Cell Longev. 2021 Oct 7;2021:3302886. doi: 10.1155/2021/3302886 (PMC8517629; doi:10.1155/2021/3302886)
Supplement: Supplementary 1 — Table S1: search strategies for PubMed and Embase. [file 3302886.f1.pdf]

Table S1. Search Strategies for Pubmed and Embase

**PubMed**	((Antioxidants OR Anti-Oxidants OR Anti Oxidants OR Antioxidant OR Anti Oxidant OR Antioxidant Activity OR Antioxidant Effect OR Anti Oxidant Effect OR Anti-Oxidant Effects OR Anti Oxidant Effects OR Antioxidant Effects) OR (Acetylcysteine OR N-Acetyl-L-cysteine OR N Acetyl L cysteine OR N-Acetylcysteine OR N Acetylcysteine OR Mercapturic Acid OR Acid, Mercapturic OR Solmucol OR Genac OR Acemuc OR Acetabs OR Acetylcystein AL OR NAC AL OR Acetylcystein Atid OR Acetylcystein Heumann OR Acetylcystein Trom OR Acetylcysteine Hydrochloride OR Hydrochloride, Acetylcysteine OR Acetylcysteine Sodium OR Monosodium Salt Acetylcysteine OR Acetylcysteine Zinc OR Zinc, Acetylcysteine OR Acetylcysteine, Monoammonium Salt OR Monoammonium Salt Acetylcysteine OR Acetyst OR Airbron OR Alveolex OR Bromuc OR Azubronchin OR Bisolvon NAC OR Broncho-Fips OR Broncho Fips OR BronchoFips OR Broncholysin OR Broncoclar OR Codotussyl OR Cystamucil OR Dampo Mucopect OR Mucopect, Dampo OR durabronchal OR Larylin NAC OR Eurespiran OR Exomuc OR Fluimucil OR NAC Zambon OR Fabrol OR Fluprowit OR Muco Sanigen OR Frekatuss OR Jenacystein OR Jenapharm OR Lantamed OR Lindocetyl OR M-Pectil OR M Pectil OR MPectil OR mentopin Acetylcystein OR Muciteran OR Mucomyst OR Acetylin OR Mucosil OR Mucosol OR Mucosolvin OR Siccoral OR Siran OR Ilube OR Hoestil OR acebraus) OR Probiotics OR (Quercetin OR Dikvertin) OR (Resveratrol OR trans-Resveratrol-3-O-sulfate OR trans Resveratrol 3 O sulfate OR SRT 501 OR SRT501 OR SRT-501 OR cis-Resveratrol OR cis Resveratrol OR trans-Resveratrol OR trans Resveratrol OR Resveratrol-3-sulfate OR Resveratrol 3 sulfate) OR Selenium OR Ozone))<br>AND<br>(Rheumatoid arthritis OR Arthritis, Rheumatoid)<br>AND<br>(random* controlled trial [pt] OR controlled clinical trial* [pt] OR randomized [tiab] OR placebo [tiab] OR drug therapy [sh] OR random* [tiab] OR trial* [tiab] OR group* [tiab])<br>NOT<br>(animals [mh] NOT humans [mh])
**EMBASE**	1 ‘Antioxidants’/exp<br>2 'Anti-Oxidants' or 'Anti Oxidants' or 'Antioxidant' or 'Anti Oxidant' or 'Antioxidant Activity' or 'Antioxidant Effect' or 'Anti Oxidant Effect' or 'Anti-Oxidant Effects' or 'Anti Oxidant Effects' or 'Antioxidant Effects'<br>3 ‘Acetylcysteine’/exp<br>4 'N-Acetyl-L-cysteine' or 'N Acetyl L cysteine' or 'N-Acetylcysteine' or 'N Acetylcysteine' or 'Mercapturic Acid' or 'Acid, Mercapturic' or 'Solmucol' or 'Genac' or 'Acemuc' or 'Acetabs' or 'Acetylcystein AL' or 'NAC AL' or 'Acetylcystein Atid' or 'Acetylcystein Heumann' or 'Acetylcystein Trom' or 'Acetylcysteine Hydrochloride' or 'Hydrochloride, Acetylcysteine' or 'Acetylcysteine Sodium' or 'Monosodium Salt Acetylcysteine' or 'Acetylcysteine Zinc' or 'Zinc, Acetylcysteine' or 'Acetylcysteine, Monoammonium Salt' or 'Monoammonium Salt Acetylcysteine' or 'Acetyst' or 'Airbron' or 'Alveolex' or 'Bromuc' or 'Azubronchin' or 'Bisolvon NAC' or 'Broncho-Fips' or 'Broncho Fips' or 'BronchoFips' or 'Broncholysin' or 'Broncoclar' or 'Codotussyl' or 'Cystamucil' or 'Dampo Mucopect' or 'Mucopect, Dampo' or 'durabronchal' or 'Larylin NAC' or 'Eurespiran' or 'Exomuc' or 'Fluimucil' or 'NAC Zambon' or 'Fabrol' or 'Fluprowit' or 'Muco Sanigen' or 'Frekatuss' or 'Jenacystein' or 'Jenapharm' or 'Lantamed' or 'Lindocetyl' or 'M-Pectil' or 'M Pectil' or 'MPectil' or 'mentopin Acetylcystein' or 'Muciteran' or 'Mucomyst' or 'Acetylin' or 'Mucosil' or 'Mucosol' or 'Mucosolvin' or 'Siccoral' or 'Siran' or 'Ilube' or 'Hoestil' or 'acebraus'<br>5 ‘Probiotics’/exp<br>6 ‘Quercetin’/exp

	7 ‘Dikvertin’
	8 ‘Resveratrol’/exp
	9 'trans-Resveratrol-3-O-sulfate' or 'trans Resveratrol 3 O sulfate' or 'SRT 501' or 'SRT501' or 'SRT-501' or 'cis-Resveratrol' or 'cis Resveratrol' or 'trans-Resveratrol' or 'trans Resveratrol' or 'Resveratrol-3-sulfate' or 'Resveratrol 3 sulfate'
	10 ‘Selenium’/exp
	11 ‘Ozone’/exp
	12 1 or 2 or 3 or 4 or 5 or 6 or 7 or 8 or 9 or 10 or 11
	13 'Arthritis, rheumatoid'/exp
	14 'Rheumatoid arthritis'
	15 13 and 14
	16 'randomized controlled trial'
	17 'single blind procedure' or 'double blind procedure'
	18 'crossover procedure'
	19 16 or 17 or 18
	20 12 and 15 and 19
